# Molecular and functional profiling of apical versus basolateral small extracellular vesicles derived from primary human proximal tubular epithelial cells under inflammatory conditions

**DOI:** 10.1002/jev2.12064

**Published:** 2021-02-16

**Authors:** Xiangju Wang, Ray Wilkinson, Katrina Kildey, Jacobus P. J. Ungerer, Michelle M. Hill, Alok K. Shah, Ahmed Mohamed, Mriga Dutt, Jeffrey Molendijk, Helen Healy, Andrew J. Kassianos

**Affiliations:** ^1^ Conjoint Internal Medicine Laboratory, Chemical Pathology Pathology Queensland Brisbane Queensland Australia; ^2^ Kidney Health Service Royal Brisbane and Women's Hospital Brisbane Queensland Australia; ^3^ Institute of Health and Biomedical Innovation Queensland University of Technology Brisbane Queensland Australia; ^4^ Faculty of Medicine University of Queensland Brisbane Queensland Australia; ^5^ QIMR Berghofer Medical Research Institute Brisbane Queensland Australia

**Keywords:** apical, basolateral, human proximal tubular epithelial cells, inflammation, small extracellular vesicles

## Abstract

Proximal tubular epithelial cells (PTEC) are central players in inflammatory kidney diseases. However, the complex signalling mechanism/s via which polarized PTEC mediate disease progression are poorly understood. Small extracellular vesicles (sEV), including exosomes, are recognized as fundamental components of cellular communication and signalling courtesy of their molecular cargo (lipids, microRNA, proteins). In this study, we examined the molecular content and function of sEV secreted from the apical versus basolateral surfaces of polarized human primary PTEC under inflammatory diseased conditions. PTEC were cultured under normal and inflammatory conditions on Transwell inserts to enable separate collection and isolation of apical/basolateral sEV. Significantly increased numbers of apical and basolateral sEV were secreted under inflammatory conditions compared with equivalent normal conditions. Multi‐omics analysis revealed distinct molecular profiles (lipids, microRNA, proteins) between inflammatory and normal conditions for both apical and basolateral sEV. Biological pathway analyses of significantly differentially expressed molecules associated apical inflammatory sEV with processes of cell survival and immunological disease, while basolateral inflammatory sEV were linked to pathways of immune cell trafficking and cell‐to‐cell signalling. In line with this mechanistic concept, functional assays demonstrated significantly increased production of chemokines (monocyte chemoattractant protein‐1, interleukin‐8) and immuno‐regulatory cytokine interleukin‐10 by peripheral blood mononuclear cells activated with basolateral sEV derived from inflammatory PTEC. We propose that the distinct molecular composition of sEV released from the apical versus basolateral membranes of human inflammatory PTEC may reflect specialized functional roles, with basolateral‐derived sEV pivotal in modulating tubulointerstitial inflammatory responses observed in many immune‐mediated kidney diseases. These findings provide a rationale to further evaluate these sEV‐mediated inflammatory pathways as targets for biomarker and therapeutic development.

## 1 INTRODUCTION

Kidney disease is a growing public health burden, affecting ∼10% of populations in industrialized countries (Gansevoort et al., 2013). Irrespective of its origins, inflammation within the tubulointerstitial compartment, the interstitial tissue adjoining the renal tubules, is considered a central determinant in most forms of kidney injury (Andrade‐Oliveira et al., 2019). Tubulointerstitial inflammation is a multifaceted biological response to injurious stimuli. In the initial injury phase, it has a protective function and is essential for tissue remodelling/repair (Rabb et al., 2016). However, if left unresolved, tubulointerstitial inflammation can drive a vicious cycle of uncontrolled tissue damage and functional decline, as observed in chronic kidney diseases (Cobo et al., 2018). Understanding the complex network of cell‐to‐cell communications between kidney tubular cells and inflammatory immune cells (e.g., monocytes, dendritic cells, T cells) is essential for developing clinical interventions to prevent this pathogenic transition to a chronic inflammatory state.

Proximal tubular epithelial cells (PTEC) of the kidney are known to respond to and mediate inflammatory processes in a wide range of kidney diseases (Schlondorff, 2008). Our group has shown that PTEC play a protective immuno‐regulatory role during the early inflammatory stages of the disease process (Kassianos et al., 2013; Sampangi et al., 2015; Sampangi et al., 2015; Wilkinson et al., 2011). This modulation of immune cell function is mediated, in part, via a repertoire of PTEC‐derived surface (programmed death ligand‐1; PD‐L1), soluble (soluble human leukocyte antigen‐G; sHLA‐G) and intracellular (indoleamine 2,3‐dioxygenase; IDO) inhibitory factors (Sampangi et al., 2015; Sampangi et al., 2015; Wilkinson et al., 2011). However, there is increasing evidence that PTEC‐derived extracellular vesicles (EV) may also play a fundamental immuno‐regulatory role under inflammatory/diseased conditions (Feigerlová et al., 2018; Wang et al., 2017).

Small extracellular vesicles (sEV), such as exosomes, are a specialized sub‐group of EV that are of endosomal origin and are released into the extracellular environment upon fusion of multivesicular bodies (MVB) with the plasma membrane (Zhang et al., 2019). They are characterized based on their size distribution (50–150 nm diameter) and expression of established sEV proteins ‐ tetraspanins (CD9, CD63, CD81), ESCRT (endosomal sorting complex required for transport) components (tumour susceptibility gene 101; TSG101 and vacuolar protein sorting 4B; VPS4B) and heat shock proteins (HSP70, HSP84) (Kalluri & Lebleu, 2020; Théry et al., 2018). sEV are secreted by many cell types under normal physiological conditions. However, in response to injurious stimuli, including inflammation, the release of sEV can be increased (Lv et al., 2019). The specific molecular cargo of secreted sEV (e.g., lipids, microRNA (miRNA), proteins) is, in turn, transferred from parental to recipient cells to mediate intercellular communication (Feigerlová et al., 2018). In addition to this functional role, the numbers and molecular content of sEV released into biological fluids (e.g., blood, urine) can be used as novel non‐invasive biomarkers of injury (Lv et al., 2019).

We have previously demonstrated that human primary PTEC produce significantly increased numbers of sEV (termed exosomes in our earlier study) under inflammatory conditions and that these sEV have a unique molecular signature with associations to inflammatory disease pathways of the kidney (Wang et al., 2017). However, PTEC are polarized epithelial cells that interact with different extracellular environments at their apical versus basolateral plasma membranes (i.e., tubular lumen/urinary space vs. tubulointerstitium). Intracellular trafficking processes within polarized epithelial cells (e.g., MVB delivery to the plasma membrane) are asymmetric. As such, there is a polarized release of sEV/exosomes within these cells, with discrete molecular cargo in sEV from apical versus basolateral surfaces (Bu et al., 2010; Chen et al., 2016; Dang et al., 2017; Sreekumar et al., 2010). This concept has yet to be investigated in human primary PTEC under normal or diseased conditions.

Here, we extend our previous findings to examine whether human primary PTEC‐derived sEV are secreted in a polarized manner under inflammatory conditions. We establish a Transwell‐based system for purifying and characterising apical versus basolateral sEV from human primary PTEC. We demonstrate that sEV with distinct molecular cargo (lipids, miRNA, proteins) are differentially secreted from the apical and basolateral membranes of inflammatory PTEC. Moreover, we present functional data suggesting basolateral sEV produced by inflammatory PTEC play a critical protective role in the initial injury phase of human kidney diseases. Our collective findings identify novel inflammatory kidney disease targets with potential for urinary biomarker development (apical sEV) and immunotherapeutic translation (basolateral sEV).

## 2 MATERIALS AND METHODS

### 2.1 Isolation and culture of human primary PTEC

Renal cortical tissue was obtained with informed patient consent from the macroscopically/microscopically healthy portion of tumour nephrectomies (Table S1), following approval by the Royal Brisbane and Women's Hospital Human Research Ethics Committee (2002/011). PTEC were purified following the method of Glynne and Evans (Glynne, 1999) and cultured in Defined Medium (DM) as previously described (Kassianos et al., 2013). DM comprised a 1:1 mixture of Dulbecco's Modified Eagle's Medium and Ham's F12 containing 15 mM HEPES buffer, 17.5 mM D‐Glucose, 2.5 mM L‐Glutamine and pyridoxine hydrochloride (Life Technologies, Grand Island, NY, USA), with the medium supplemented with epidermal growth factor (10 ng/ml), insulin (10 μg/ml), transferrin (5 μg/ml), selenium (5 ng/ml), hydrocortisone (36 ng/ml), triiodothyronine (4 pg/ml) (all from Sigma‐Aldrich, St Louis, MO, USA) and penicillin (50 U/ml) and streptomycin (50 mg/ml) (both from Life Technologies). PTEC were used in experiments at passage 4.

### 2.2 Treatment of human primary PTEC on permeable membranes

Apical versus basolateral sEV production by PTEC was examined using Transwell^®^ plates (Corning, Cambridge, MA, USA). Human primary PTEC were seeded onto 6‐well transparent polyester Transwell inserts (0.4 μm pore size, 24 mm diameter, 4.67 cm^2^ surface area) at a concentration of 1.2 × 10^5^ cells/cm^2^ in DM (2.5 ml volume in the upper compartment). DM alone (3.1 ml) was added to the lower compartment. PTEC were grown to confluence as confirmed in permeability studies described below.

Once monolayer integrity was confirmed, the DM in the upper and lower compartments was exchanged with fresh DM for normal control PTEC and fresh DM supplemented with 100 ng/ml interferon (IFN)‐γ and 20 ng/ml tumour necrosis factor (TNF)‐α (both from R&D Systems, Minneapolis, MN, USA) for inflammatory PTEC and then further cultured for 72 h. PTEC culture medium was subsequently harvested from the upper (apical) compartment (2.5 ml for each Transwell) and lower (basolateral) compartment (3.1 ml for each Transwell). Individual collections of apical and basolateral media for each culture condition from each PTEC donor were pooled for downstream sEV isolation.

### 2.3 Permeability studies

The permeability of PTEC monolayers was measured using fluorescein isothiocyanate (FITC)‐Dextran (molecular mass 70 kDa; Sigma‐Aldrich) as previously described (Whitin et al., 2002). PTEC were cultured on Transwell inserts as described above. Transwell inserts and culture wells were washed twice with phosphate‐buffered saline (PBS; Life Technologies, Grand Island, NY, USA). A 1 ml solution of 100 μg/ml FITC‐Dextran in transport buffer [10 mM HEPES buffer in Hanks’ buffered salt solution (HBSS) containing calcium and magnesium; both from Invitrogen/Thermo Fisher Scientific, Carlsbad, CA, USA] was added to the apical Transwell insert. Transport buffer alone (1 ml) was added to the lower (basolateral) chamber. After 1 h, the basolateral solution was collected. The fluorescent signals of the basolateral samples (test), transport buffer (blank) and 1 μg/ml FITC‐Dextran solution (FDS) were measured in a Synergy H4 plate reader (excitation wavelength 485 nm; emission wavelength 535 nm; Biotek, Winooski, VT, USA). Percent permeability was calculated as 100 x (basolateral sample‐blank)/(FDS‐blank). A stringent threshold of ≤ 2% permeability was required for continuation of experiments.

### 2.4 Immunofluorescent studies

Cellular distribution and maintenance of PTEC polarity were examined by immunofluorescent (IF) staining. PTEC monolayers on Transwell inserts were fixed with 2% paraformaldehyde (Sigma‐Aldrich) at room temperature for 5 min, followed by permeabilisation with 0.2% Triton X‐100 (Sigma‐Aldrich) at room temperature for 15 min and a protein block with 1% Bovine Serum Albumin (BSA) (Sigma‐Aldrich) at room temperature for 30 min. PTEC were probed with primary antibodies against Zonula occludens‐1 (ZO1) (Rabbit monoclonal IgG; Cell Signaling Technology, Danvers, MA, USA), E‐Cadherin (Mouse monoclonal IgG1; Abcam, Cambridge, MA, USA), β‐tubulin (Rabbit polyclonal IgG; Abcam) and multidrug resistance‐associated protein (MRP)‐4 (Mouse monoclonal IgG1; Abcam) or isotype‐matched control antibodies at room temperature for 2 h. Fluorescent detection was obtained by secondary incubation with Alexa Fluor‐488 anti‐rabbit IgG and Alexa Fluor‐555 anti‐mouse IgG (both from Life Technologies) at room temperature for 40 min. Nuclei were stained with DAPI (Invitrogen). Membranes were cut from Transwell inserts, mounted on slides and coverslipped in fluorescence mounting medium (Agilent Technologies, Santa Clara, CA, USA). A Zeiss 780 NLO confocal microscope (Carl Zeiss, Hamburg, Germany) was used for fluorescence microscopy. Image acquisition and analysis were performed using ZEN software (Carl Zeiss).

### 2.5 sEV isolation

PTEC culture medium was centrifuged at 300 x *g* for 10 min at 4°C and filtered through a 0.22 μm filter (Merck Millipore, Bayswater, Victoria, Australia) to remove contaminating apoptotic bodies and cell debris. The clarified supernatant was transferred to an Amicon^®^ Ultra‐15 100,000 Da device (Merck Millipore) and concentrated to a 500 μl volume by centrifugation at 3500 x *g* at 4°C. The concentrated supernatant was loaded onto a qEV size exclusion column (Izon Science Ltd, Christchurch, New Zealand), with sEV isolation performed as per the manufacturer's instructions. Briefly, the concentrated supernatant was overlaid on the qEV exclusion column followed by elution with PBS. The flow‐through was eluted in 500 μl fractions, with sEV fractions 5–11 collected and pooled. sEV were then ultracentrifuged at 100,000 x *g* for 1.5 h at 4°C to concentrate samples. The resulting pellets were resuspended in 100 μl PBS. The total number and size distribution of sEV was analysed with tuneable resistive pulse sensing (TRPS) (qNano, Izon Science Ltd) following the method of Wang et al. (Wang et al., 2017). Briefly, total particles/Transwell were calculated as: total number of sEV/volume of the initial pooled PTEC culture supernatant x Transwell chamber volume (TCV); where TCV is 2.5 ml for apical (upper chamber) samples and 3.1 ml for basolateral (lower chamber) samples. Total particles/Transwell were then normalized to total particles from equivalent 1 cm^2^ area of confluent Transwell monolayer (total particles/cm^2^), based on a surface area of 6‐well Transwell inserts of 4.67 cm^2^.

### 2.6 Electron microscopy

Purified sEV were applied on a Formvar‐coated and carbon stabilised copper grid and stained with 2% aqueous uranyl acetate. Samples were examined using a JEM 1011 transmission electron microscope operated at 80 kV and equipped with a digital camera.

### 2.7 Western blotting

Cells and sEV were lysed with Pierce™ RIPA lysis buffer (Pierce Protein Biology/Thermo Fisher Scientific, Waltham, MA, USA) containing protease inhibitor (Sigma‐Aldrich) and protein content determined using the BCA protein assay (Pierce Protein Biology/Thermo Fisher Scientific). Polyacrylamide gel electrophoresis (PAGE) was performed using standard reagents from Invitrogen/Thermo Fisher Scientific. Samples were denatured for 5 min at 95°C, loaded onto Bolt™ 4–12% Bis‐Tris Plus Gels, run at 200 V for 28 min and transferred to a nitrocellulose membrane at 10 V for 60 min. Membranes were blocked for 1 h at room temperature using Intercept^®^ (TBS) blocking buffer (LI‐COR, Lincoln, NE, USA) and subsequently probed with primary antibodies overnight at 4°C, including CD63 (Mouse monoclonal IgG1, Clone Ts63; Invitrogen/Thermo Fisher Scientific), CD9 (Mouse monoclonal IgG1, Clone Ts9; Invitrogen/Thermo Fisher Scientific), CD81 (Mouse IgG1, Clone M38; Invitrogen/Thermo Fisher Scientific) and Calnexin (Mouse monoclonal IgG1, Clone AF18; Santa Cruz, Dallas, TX, USA). Proteins were visualized with IRDye 800CW goat anti‐mouse (LI‐COR) using the Odyssey CLX (LI‐COR). Quantitative analysis of protein intensities was performed using Image Studio 5.2 software (LI‐COR).

### 2.8 miRNA analysis

Total RNA isolation, miRNA‐seq library construction, sequencing and analysis were performed at Beijing Genomics Institute (BGI) (Shenzhen, China). Briefly, total RNA was extracted from sEV. Small RNA fragments of 18–30 nucleotides were isolated and purified from total RNA by PAGE. Small RNA were sequentially ligated to 3′ and 5′ adaptors and converted to complementary DNA (cDNA) by reverse transcription polymerase chain reaction (RT‐PCR). PCR amplification was performed to enrich for cDNA with both 3′ and 5′ adaptors, followed by library fragment selection to eliminate primer‐dimers and other by‐products. Circularization was performed to generate single‐stranded DNA circles. Circular single‐stranded libraries were subsequently used as a template for rolling circle amplification to form DNA nanoballs. The DNA nanoballs were loaded onto a sequencing flow cell and then processed for 50 bp paired‐end sequencing on the BGISEQ‐500 platform.

Analysis of miRNA was performed after removal of low quality and contaminant tags. Remaining clean reads were mapped to reference genome and other miRNA databases using Bowtie (Langmead et al., 2009). Novel miRNA were predicted using miRDeep2 (Friedländer et al., 2008). Libraries were normalized using the TPM (transcripts per million) method. Expression levels of miRNA were considered as statistically significantly differentially expressed when the log_2_ fold change (FC) was ≥1, and the adjusted *P*‐value (accounting for multiple testing false discovery rate (FDR) correction) was ≤0.001.

### 2.9 Lipid and protein extraction

Purified sEV were subjected to single phase solvent extraction using 100% v/v methanol containing 50 μg/ml butylated hydroxytoluene (BHT). Briefly, ten volumes of cold methanol (+50 μg/ml BHT) was added to each sample, followed by incubation at ‐20°C for 24 h. Samples were then centrifuged at 16,000 x *g* for 15 min at 4°C. Supernatants were collected as the lipid‐rich extract, with protein recovered in the pellets. The lipid‐rich extracts were further processed by adjusting the volume to 200 μl methanol (+50 μg/ml BHT) and the addition of 750 μl methyl‐tert‐butyl ether (MTBE), based on the method by Matyash et al. (Matyash et al., 2008). Each tube was briefly vortex mixed (30 s) and shaken for 10 min on a tube rotator at 4°C. MilliQ water (188 μl) was added, followed by 30 s of vortex mixing and centrifuging at 15,000 x *g* for 15 min to induce a biphasic separation. After collecting 700 μl of the upper phase (MTBE), the lipid‐rich phase was evaporated to dryness under a gentle stream of nitrogen and then reconstituted in 30 μl of a methanol (containing 50 μg/ml BHT)/toluene (90%/10%, v/v) mixture for liquid chromatography‐mass spectrometry (LC‐MS) analysis described below.

### 2.10 Proteomic analysis

The concentration for each apical sEV protein pellet was determined by Pierce BCA protein assay for downstream in‐solution digestion. For basolateral samples, the entire protein pellet was subjected to proteolytic digestion due to their low yield. Apical (2 μg starting amount) and basolateral (variable starting amounts) sEV protein samples were reduced with 10 mM tris(2‐carboxyethyl)phosphine (Merck Millipore) at 60°C for 30 min, followed by alkylation with 40 mM 2‐chloroacetamide (Merck Millipore) at room temperature for 30 min in the dark. Digestion of proteins with sequencing grade porcine trypsin (1:30 enzyme:protein ratio; Promega, Madison, WI, USA) was then performed at 37°C for 18 h. Digestion was stopped by acidification to a final concentration of 1% v/v formic acid (FA) in H_2_O (Merck Millipore). The digested samples were then dried in a vacuum and resuspended in 12 μl of 0.1% v/v FA in H_2_O prior to tandem liquid chromatography‐mass spectrometry (LC‐MS/MS).

Tryptic peptides (1 μg for apical samples; variable for basolateral samples) were analysed using a TripleTOF^®^ 5600^+^ mass spectrometer (SCIEX, Framingham, MA, USA) fitted with a nanospray ion source, coupled to an Eksigent UltraNano 1D Plus liquid chromatography system (SCIEX). 10 μl of peptide samples were loaded onto an Eksigent ChromXP‐C18 trap column (10 × 0.3 mm, 17 μm) and separated on an Eksigent ChromXP‐C18 analytical column [(0.075 × 150 mm, 11 μm] over a 95 min gradient at 0.25 μl/min flow rate.

The peptides were eluted using Buffer A (0.1% v/v FA in H_2_O) and Buffer B (0.1% v/v FA in acetonitrile), over the specified linear gradient for Buffer B (2–10% at 2 min, 10–40% at 60 min, 40–50% at 65 min, 50–95% at 75 min and maintained at 95% for an additional 15 min). The nanosource parameters were set as follows: gas 1 = 5 psi, curtain gas = 25 psi, interface heater temperature (IHT) = 150°C and ion spray floating voltage (ISVF) = 2200 V. Information dependent acquisition (IDA) was performed using the Top 18 method. The MS1 spectra were acquired in positive polarity within the mass range = m/z 300–1800 Da, with the accumulation time = 200 ms. The MS/MS spectra were acquired using collision induced dissociation (CID) within the mass range = m/z 100–2000 Da, with the following parameters: charge states +2 to +4, accumulation time = 100 ms and dynamic exclusion = 15 s.

The acquired raw ion spectra were searched for protein IDs against the reviewed UniProt human proteome database (20,359 proteins, accession date 01/07/2018) using MaxQuant version 1.6.0.16 (Cox & Mann, 2008). MaxQuant parameters were set as follows: Digestion = trypsin, with two missed cleavages; fixed modification was set to carbamidomethyl; variable modifications were set to N‐terminal acetylation and methionine oxidation; label free quantification (LFQ) was enabled with minimum ratio count set to 2; unique and razor peptides were used for protein identification; match between runs was set as TRUE; and FDR was set at 0.01.

Identified proteins were filtered according to unique peptides (≥2) and Score (*>* 5), and then according to missing values, where proteins were only kept if they were detected in all samples of one or more comparison groups. Data was normalized by total protein intensity and remaining missing values imputed using two techniques: i) proteins that were exclusively detected in one group, and were missing in other groups because of low abundance, were imputed from a normal distribution centred at minimum intensity; and (ii) proteins missing in multiple samples in different groups were assumed to be caused by interference or ion suppression, and were imputed using localized least square regression as described in (Valikangas et al., 2018). Imputations for apical versus basolateral sEV samples were performed separately due to the large difference in their proteomes. Quantitative differential analysis of log_2_ transformed data was performed using the limma R‐package to identify significant proteins (FDR adjusted *P*‐value *<* 0.05, log_2_FC *>* 1.5).

### 2.11 Pathway analysis

Annotations for significantly dysregulated miRNA/proteins were obtained using the Ingenuity Pathway Analysis (IPA) program (Qiagen, Melbourne, Victoria, Australia) following the core analysis workflow using standard parameters: stringent filter for molecules and relationship and no protein fold change cut‐off applied. Readouts included the “Disease and Biological Function”, “Tox” clustering and “Networks Function” pathway analyses.

### 2.12 Lipidomics

Targeted lipidomics experiments were performed using an Agilent Technologies 1290 Infinity II UHPLC system with an Agilent ZORBAX eclipse plus C18 2.1 × 100 mm; 1.8 μm column, coupled online to an Agilent 6470 Triple Quadrupole Mass spectrometer operated in positive ionization mode. Chromatographic separation and column compartment parameters were as described by Huyhn et al. (Huynh et al., 2019). The source nitrogen gas temperature was set to 175°C at a flow rate of 11 L/min, and the sheath gas temperature set to 250°C at a flow rate of 10 L/min. The capillary voltage was set to 3500 V for positive and the nebulizer operated at 20 psi. Isolation widths for the quadrupoles Q1 and Q3 were set to “unit” resolution. Lipids were measured in dynamic MRM mode using previously published transitions (Huynh et al., 2019).

The acquired lipidomics data was processed in Skyline for peak integration (Maclean et al., 2010) and data for 77 lipids was exported. Lipidomics data analysis was performed using the lipidr R package (Mohamed et al., 2020). Briefly, raw data was log_2_ transformed and normalized using the probabilistic quotient normalization method (Dieterle et al., 2006). Quantitative analysis was performed, with differentially expressed lipids with a log_2_FC ≥1 and the FDR adjusted *P*‐value < 0.05 considered significant. Lipid set enrichment analyses were performed to identify lipid classes significantly affected by the treatments.

### 2.13 Human PBMC isolation

Leukocyte‐rich buffy coats were obtained from healthy blood donors (Australian Red Cross Blood Service). Peripheral blood mononuclear cells (PBMC) were isolated from buffy coats using SepMate™ isolation tubes (Stemcell Technologies, Vancouver, Canada) and Ficoll‐Paque™ Plus density gradient centrifugation (GE Healthcare, Uppsala, Sweden). For downstream sEV uptake/activation experiments, isolated PBMC were resuspended in Complete Medium (CM) consisting of RPMI 1640, supplemented with 10% heat‐inactivated fetal bovine serum (FBS), 100 U/ml penicillin, 100 μg/ml streptomycin, 2 mM L‐glutamine, 1 mM sodium pyruvate, 0.1 mM non‐essential amino acids, 10 mM HEPES buffer solution (all from Invitrogen) and 50 μM 2‐mercaptoethanol (Sigma‐Aldrich).

### 2.14 sEV uptake

For uptake experiments, isolated sEV were fluorescently labelled with 5‐(and 6)‐Carboxyfluorescein diacetate succinimidyl ester (CSFE) (Biolegend, San Diego, CA, USA). sEV (1–5 × 10^9^ sEV in 150 μl PBS) were labelled with CFSE (150 μl of 40 μM stock) at 37°C in the dark for 20 min. Excess CFSE dye was removed using an Exosome Spin Column (MW3000) (Life Technologies) according to manufacturer instructions, with labelled sEV eluted in PBS.

PBMC were incubated with CFSE‐labelled sEV (cell:sEV ratio of 1:1000) at 37°C for 2 h. Following incubation, cells were washed twice in ice‐cold FACS buffer [0.5% BSA and 0.02% sodium azide (Sigma‐Aldrich) in PBS] and then stained with LIVE/DEAD^®^ Fixable Near‐IR Dead Cell reagent (to exclude dead cells) (Life Technologies), CD3‐Brilliant Violet 650 (Biolegend) and CD14‐Alexa Fluor 700 (Biolegend) or appropriate isotype controls for flow cytometric assessment of sEV uptake. Conventional flow cytometry was performed on an LSR Fortessa (BD Biosciences, San Jose, CA, USA), with data analysed using FlowJo software (TreeStar, Ashland, OR, USA). Imaging flow cytometry was performed on an ImageStream^®X^ Mark II (Luminex, Austin, TX, USA), with data analysis using IDEAS version 6.2 (Luminex). Where indicated, PBMC were pre‐incubated prior to sEV co‐culture with an inhibitor of actin polymerization, cytochalasin D (CCD; 25 μg/ml final concentration; Sigma‐Aldrich).

### 2.15 sEV activation of human PBMC

PBMC were cultured in 96‐well plates in CM for 24 h in the presence of sEV (cell:sEV ratio of 1:1000; 200 μl final volume). PBMC culture supernatants were harvested and cytokine levels were determined using the LEGENDplex™ Human Inflammation Panel multiplex bead‐based assay (Biolegend) according to the manufacturer's instructions. Cytokine values were normalized to an equivalent PBMC concentration of 1.275 × 10^6^ cells/ml.

### 2.16 Statistics

Statistical tests for sEV enumeration, Western blotting and functional assays were performed using Prism 7.0 analysis software (GraphPad Software, La Jolla, CA, USA). Comparisons between paired groups were performed using a Wilcoxon matched‐pairs signed rank test. *P* values ≤0.05 were considered statistically significant.

## 3 RESULTS

### 3.1 Establishment of a Transwell model to examine polarized sEV production by human primary PTEC

**FIGURE 1:**
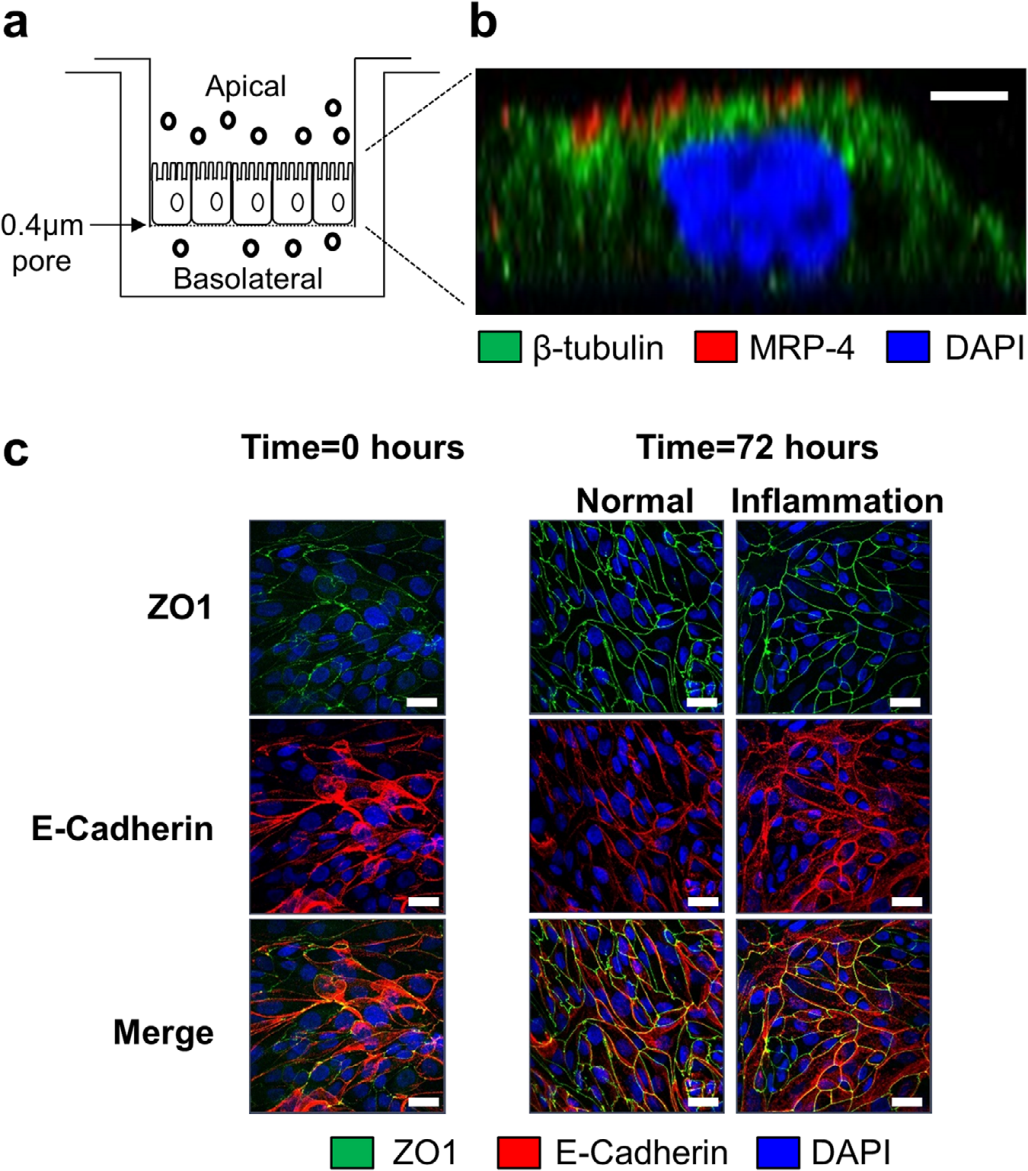
**Establishment of a Transwell model to examine polarized sEV production by human primary PTEC**. (a) Human primary PTEC were seeded onto Transwell inserts (0.4 μm pore size) and grown to confluence. The defined medium (DM) was subsequently exchanged with fresh DM for normal control PTEC and fresh DM supplemented with 100 ng/ml IFN‐γ and 20 ng/ml TNF‐α for inflammatory PTEC and then further cultured for 72 h. PTEC culture medium was then harvested from the upper (apical) compartment and lower (basolateral) compartment for downstream sEV isolation. (b) Immunofluorescent microscopy of inflammatory PTEC monolayers on Transwell inserts (lateral projection) stained for β‐tubulin (green), MRP‐4 (red) and DAPI (blue). Scale bar represents 2 μm. One representative of three PTEC donor experiments. Equivalent staining profile was observed for normal control PTEC monolayers. (c) Immunofluorescent staining of PTEC monolayers on Transwell inserts for ZO1 (green), E‐Cadherin (red) and DAPI (blue) at time = 0 h and following 72 h culture under normal and inflammatory conditions. Scale bars represents 20 μm. One representative of two PTEC donor experiments.

We established an *in vitro* model for investigating sEV produced from the apical versus basolateral surfaces of polarized human primary PTEC (Figure 1a). Human PTEC were cultured to confluency on 0.4 μm Transwell inserts to generate an impermeable cell monolayer, thus enabling the characterisation of sEV produced from the apical plasma membrane into the upper compartment and basolateral plasma membrane into the lower compartment. The impermeability of confluent monolayers at this time‐point (time = 0 h) was confirmed by assessing the diffusion of FITC‐Dextran from the upper (apical) to the lower (basolateral) chamber. The confluent PTEC monolayer was shown to effectively eliminate diffusion from the apical to the basolateral compartment, with a percent permeability of 0.55 ± 0.14% measured at time = 0 h (Table 1).

**TABLE 1:** Apical‐to‐basolateral diffusion (expressed as percent permeability; % permeability) for Transwells with confluent PTEC monolayers at time = 0 h and following culture under normal (control) or inflammatory (diseased) conditions (time = 72 h)

Condition	% Permeability (*n* = 8)
*Time = 0 hours*	
Confluent monolayer	0.55 *± 0.14*
*Time = 72 hours*	
Normal	0.75 ± 0.05
Inflammation	0.22 ± 0.05

Results represent mean ± SEM of eight individual PTEC donor experiments.

Human PTEC monolayers were subsequently cultured under normal control or inflammatory diseased conditions for 72 h. The PTEC monolayer remained impermeable under both experimental conditions at this time‐point, with the percent permeability measured at 0.75 ± 0.05% under normal conditions and 0.22 ± 0.05 under inflammatory conditions (Table 1). The formation of a restrictive PTEC monolayer was also confirmed by the expression of tight junction protein Zonula occludens‐1 (ZO1) and intercellular junction marker E‐cadherin at cell‐cell contact points in PTEC monolayers at time = 0 h and following 72 h culture under normal and inflammatory conditions (Figure 1c).

The polarity of PTEC monolayers following *in vitro* treatment was also examined. Immunofluorescence (IF) staining of confluent monolayers cultured under normal and inflammatory conditions showed expression of apical protein MRP‐4 to be retained on the upper surface and facing the top compartment, indicating a maintenance of polarity (Figure 1b and Figure S1a,b). We used this Transwell model to subsequently examine the polarized secretion of sEV by human primary PTEC.

### 3.2 Significantly elevated sEV production by human primary PTEC under inflammatory diseased conditions

**FIGURE 2:**
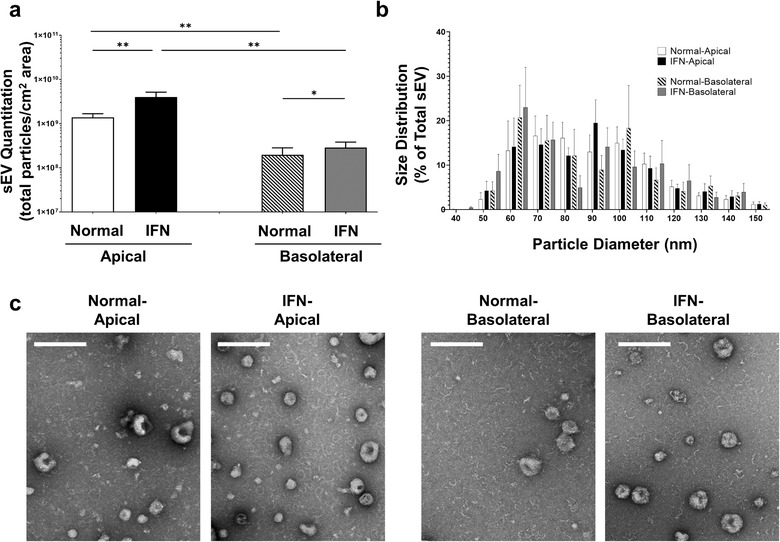
**Significantly elevated sEV production by human primary PTEC under inflammatory conditions**. (a) Apical and basolateral sEV numbers (normalized to total particles from equivalent 1 cm^2^ area of confluent Transwell monolayer) produced by human primary PTEC under normal and inflammatory (IFN) culture conditions. Results represent mean ± SEM of eight individual PTEC donor experiments. *P < 0.05, **P < 0.01, Wilcoxon matched‐pairs signed‐rank test. (b) Equivalent size distribution of apical and basolateral sEV derived from primary human PTEC under normal and inflammatory (IFN) conditions; analysed with tunable resistive pulse sensing (TRPS) using a NP100 nanopore at a 45 mm stretch. Size distribution data represent the proportion of total sEV particles for each condition, with the mean ± SEM of eight individual PTEC donor experiments presented. (c) Electron microscopy images of apical and basolateral sEV purified from primary human PTEC under normal and inflammatory (IFN) culture conditions. Scale bar represents 200 nm. One representative of two PTEC donor experiments.

PTEC culture media were collected from the apical and basolateral compartments for both normal and inflammatory conditions. The individual collections of apical and basolateral media for each culture condition from each PTEC donor were pooled for sEV isolation using commercial qEV size exclusion columns. Using TRPS analysis, we then measured the total numbers and size of isolated sEV. The numbers of apical and basolateral sEV isolated from inflammatory conditions (normalized to total particles from equivalent 1 cm^2^ area of confluent Transwell monolayer) were significantly elevated compared with equivalent sEV from normal conditions (Figure 2a). Of note, production of sEV from the apical membrane was significantly more abundant compared with the basolateral membrane for both normal and inflammatory conditions. There were no significant differences in size distributions, with all populations exhibiting the equivalent sEV size profile of 50–150 nm (Figure 2b). Electron microscopy analysis of purified sEV was also undertaken, further confirming the equivalent size distribution profiles of apical/basolateral sEV under normal and inflammatory conditions (Figure 2c).

**FIGURE 3:**
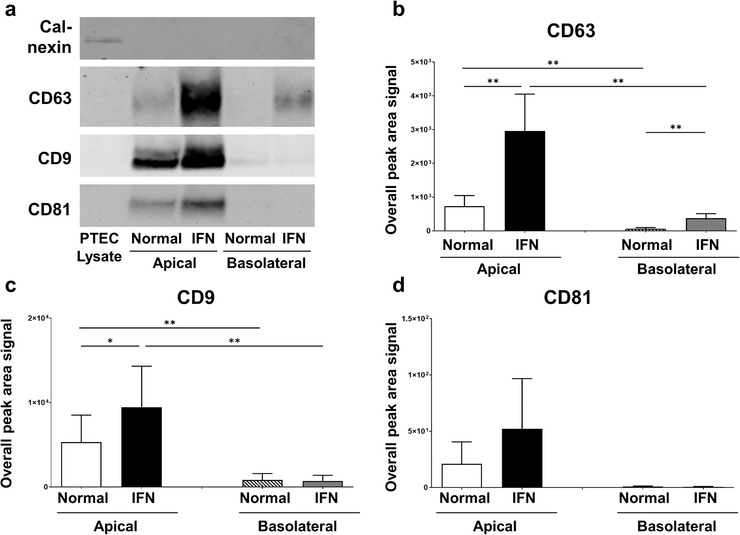
**Human primary PTEC cultured under inflammatory conditions produce increased numbers of sEV – defined by CD63/CD9/CD81 content**. (a) Western Blot for calnexin, CD63, CD9, and CD81 in PTEC lysate (2 μg total protein) and purified apical and basolateral sEV lysates (particles from equivalent 1 cm^2^ area of confluent PTEC monolayer) produced by human primary PTEC under normal and inflammatory (IFN) culture conditions. Representative data from eight individual PTEC donor experiments for calnexin, CD63 and CD9 and three individual PTEC donor experiments for CD81 are presented. (b‐d) Overall peak area signal for CD63 (b), CD9 (c), and CD81 (d) from purified apical and basolateral sEV lysates (particles from equivalent 1 cm^2^ area of confluent PTEC monolayer) produced by human primary PTEC under normal and inflammatory (IFN) culture conditions. Results represent mean ± SEM of eight individual PTEC donor experiments for CD63 and CD9 and three individual PTEC donor experiments for CD81. *P < 0.05, **P < 0.01, Wilcoxon matched‐pairs signed‐rank test.

Western blot analysis of PTEC lysate and isolated sEV (particles from equivalent 1 cm^2^ area of confluent PTEC monolayer) was subsequently performed. The endoplasmic reticulum protein Calnexin was present in PTEC lysate, but absent from all sEV populations, confirming the purity of the sEV preparations. In line with our quantitative TRPS data, Western blot analysis showed significantly increased expression of canonical sEV protein CD63 from inflammatory conditions compared with normal conditions for both apical and basolateral sEV (Figure 3a,b). Expression levels of sEV markers CD9 and CD81 were similarly elevated (significantly for CD9) for apical sEV from inflammatory conditions compared with normal conditions (Figure 3a,c,d). However, expression of these two markers in basolateral sEV was either not detectable or low (Figure 3a), preventing robust quantitative comparisons between basolateral sEV from normal and inflammatory conditions for CD9 and CD81 to be performed.

### 3.3 Significant enrichment of ceramides in sEV produced by human primary PTEC under inflammatory diseased conditions

We next examined the molecular content of purified sEV populations. Targeted lipidomics was performed on sEV from four individual donor PTEC experiments. The targeted lipidomics data was processed using Skyline, and included 77 lipid species across 13 classes of lipids. Differential analysis was performed after data were log_2_ transformed and normalized using the probabilistic quotient normalization method. Quantitative analysis identified significant differences (log_2_ fold change ≥1; FDR adjusted *P*‐value < 0.05) in lipid species (Table S2) and classes (Table 2) in apical and basolateral sEV isolated from inflammatory conditions compared with equivalent sEV from normal conditions. In particular, ceramides were significantly elevated in apical and basolateral inflammatory sEV. This increased abundance of ceramides, established as crucial cell signalling molecules during inflammatory responses (Gomez‐Muñoz et al., 2016), suggests that PTEC‐derived inflammatory sEV are active participants in the diseased kidney microenvironment.

**TABLE 2:** Significantly differentially expressed sEV lipid classes

Lipid class	Log_2_ fold change	FDR‐adjusted *P*‐value
*IFN‐apical versus normal‐apical*		
HexCer	−0.7236	0.0424
Cer	1.3671	0.0033
*IFN‐basolateral versus normal‐basolateral*		
Cer	1.5308	0.0044

### 3.4 Unique miRNA profile of sEV produced by human primary PTEC under inflammatory diseased conditions

We next examined the miRNA content of purified sEV. Libraries were normalized using the TPM method to enable differential analysis between inflammatory and normal sEV (log_2_ fold change ≥1), with additional high‐stringency filtering to exclude miRNA species not commonly differentially expressed between conditions across all four individual donor PTEC experiments. Quantitative analysis of retained molecules identified 23 novel miRNA as significantly differentially expressed (FDR adjusted *P*‐value ≤ 0.001) between inflammatory and normal sEV from the apical membrane (one down‐regulated; 22 up‐regulated) and three to be significantly differentially expressed between inflammatory and normal sEV from the basolateral membrane (all up‐regulated) (Table 3 and Table S3). Of defined miRNA identified, 26 were significantly differentially expressed between inflammatory and normal sEV from the apical membrane (12 down‐regulated; 14 up‐regulated), with four molecules significantly differentially expressed between inflammatory and normal sEV from the basolateral membrane (three down‐regulated; one up‐regulated) (Table 3 and Table S4).

**TABLE 3:** Numbers of significantly differentially expressed [upregulated (↑) and downregulated (↓)] sEV novel and defined miRNA

	Novel miRNA		Defined miRNA	
	↓	↑	↓	↑
IFN‐apical versus normal‐apical	1	22	12	14
IFN‐basolateral versus normal‐basolateral	0	3	3	1

### 3.5 Molecular profile (miRNA/protein) of sEV produced by human primary PTEC under diseased conditions is associated with inflammatory/immunological pathways

Analysis of the protein content of sEV from three individual donor PTEC experiments identified a total of 625 proteins, of which 463 proteins were subjected to normalization/differential analysis after filtering. Of these 463 proteins, established sEV tetraspanins (CD63, CD9, CD81), ESCRT components (TSG101, ALIX, VPS4B) and heat shock proteins (HSP70, HSP84) were identified across all samples, further demonstrating the nature and purity of our sEV preparations. Subsequent quantitative analysis identified 22 proteins as significantly differentially expressed (log_2_FC *>* 1.5; FDR adjusted *P*‐value *<* 0.05) between inflammatory and normal sEV from the apical membrane (two down‐regulated; 20 up‐regulated) and eleven to be significantly differentially expressed between inflammatory and normal sEV from the basolateral membrane (all up‐regulated) (Table 4).

**TABLE 4:** Significantly differentially expressed sEV proteins

Protein	Gene symbol	Log_2_ fold change	FDR‐adjusted *P*‐value
*IFN‐apical versus normal‐apical*			
60S ribosomal protein L27	RPL27	−5.2203	0.0252
V‐set domain‐containing T‐cell activation inhibitor 1	VTCN1	−2.9449	0.0460
Protein lin‐7 homolog C	LIN7C	2.3728	0.0382
HLA class I histocompatibility antigen, A‐24 alpha chain	HLA‐A	3.3523	0.0302
TNFAIP3‐interacting protein 1	TNIP1	3.4011	0.0360
Laminin subunit alpha‐3	LAMA3	3.4341	0.0350
Beta (β)‐2‐microglobulin	B2M	3.4718	0.0168
Interferon‐induced transmembrane protein 1	IFITM1	4.2395	0.0351
Tumour necrosis factor alpha‐induced protein 2	TNFAIP2	4.5402	0.0151
Myosin‐10	MYH10	4.6153	0.0191
Serum amyloid A‐1 protein	SAA1	4.6337	0.0099
Intercellular adhesion molecule 1	ICAM1	4.9056	0.0168
C‐X‐C motif chemokine 10	CXCL10	5.1736	0.0264
Alcohol dehydrogenase [NADP(+)]	AKR1A1	5.3855	0.0302
Hyaluronan and proteoglycan link protein 3	HAPLN3	5.4842	0.0056
Guanylate‐binding protein 5	GBP5	6.5357	0.0287
Indoleamine 2,3‐dioxygenase 1	IDO1	6.5417	0.0151
Tryptophan–tRNA ligase, cytoplasmic 1	WARS	6.6389	0.0056
Interferon‐induced guanylate‐binding protein 1	GBP1	7.1445	0.0039
Tumour necrosis factor receptor superfamily member 5	CD40	7.3977	0.0072
ADP‐ribosyl cyclase/cyclic ADP‐ribose hydrolase 1	CD38	7.7056	0.0039
Tumour necrosis factor alpha‐induced protein 3	TNFAIP3	7.9718	0.0351
*IFN‐basolateral versus normal‐basolateral*			
Complement C3	C3	4.1754	0.0322
Intercellular adhesion molecule 1	ICAM1	4.3344	0.0257
Bone marrow stromal antigen 2	BST2	4.4433	0.0185
Destrin	DSTN	5.0007	0.0068
IST1 homolog	IST1	5.1550	0.0257
Hyaluronan and proteoglycan link protein 3	HAPLN3	5.4818	0.0189
Pituitary tumour‐transforming gene 1 protein‐interacting protein	PTTG1IP	5.6662	0.0080
ADP‐ribosyl cyclase/cyclic ADP‐ribose hydrolase 1	CD38	5.9300	0.0080
Laminin subunit beta‐2	LAMB2	5.9880	0.0185
Secreted and transmembrane protein 1	SECTM1	6.3891	0.0068
C‐C motif chemokine 5	CCL5	7.2590	0.0013

**FIGURE 4:**
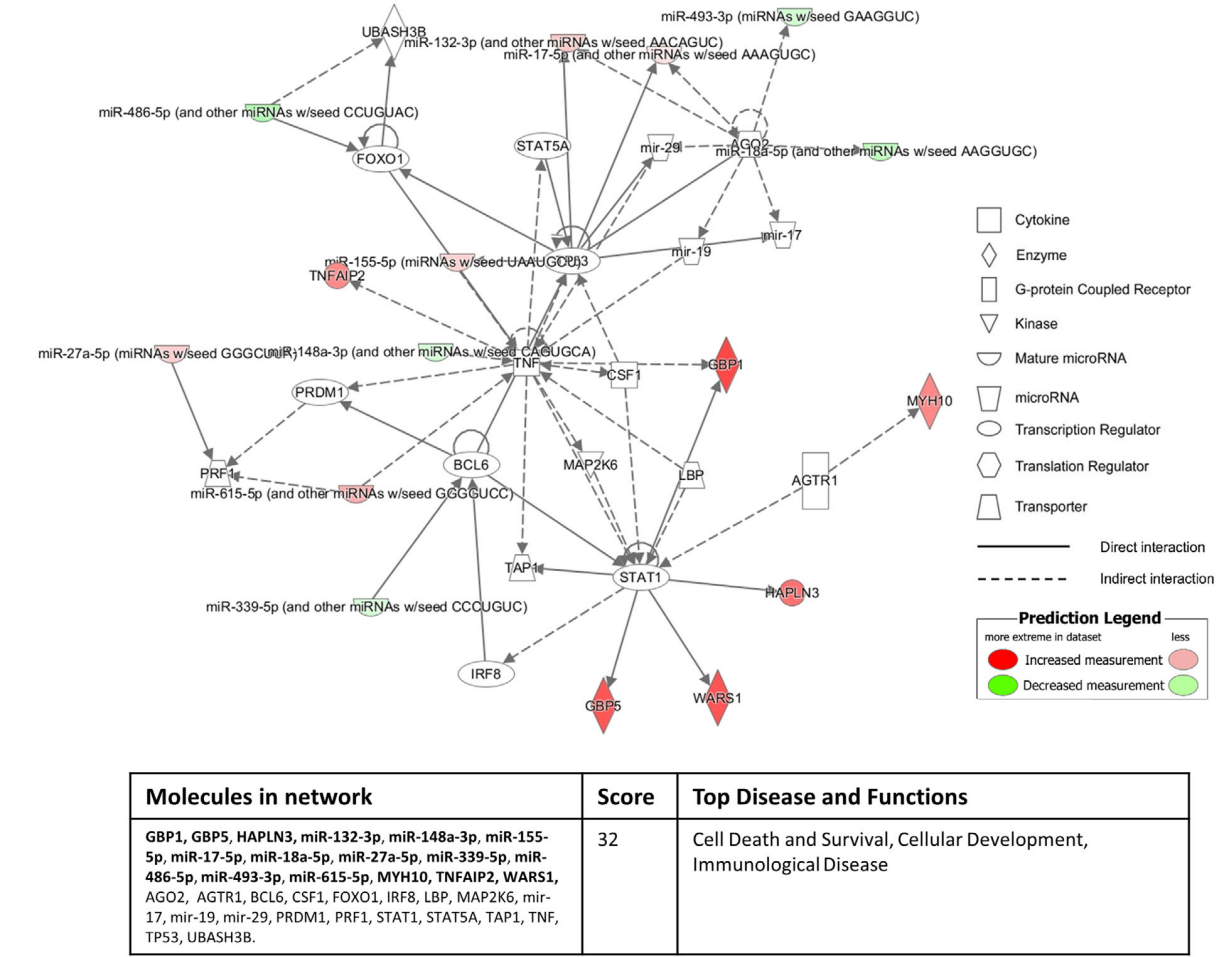
**Sixteen out of the 48 significantly differentially expressed apical sEV molecules (defined miRNA/proteins) derived from inflammatory culture conditions compared to normal culture conditions associate with a cell death and survival/immunological disease pathway identified by the “Networks Function” of IPA**. The red colour intensity indicates amount of up‐regulation within the network and the green colour intensity indicates amount of down‐regulation within the network. Molecules highlighted in bold within the data table are the 16 significantly differentially apical sEV miRNA/proteins.

To investigate the functional associations of miRNA/proteins identified as significantly differentially expressed between inflammatory diseased and normal control conditions, we conducted unbiased pathway analyses using the “Disease and Biological Function” clustering function of the Ingenuity Pathway Analysis (IPA) program with a stringent *P*‐value cut‐off of < 1e‐4. When mapping the significantly differentially expressed defined miRNA (26) and proteins (22)  of inflammatory versus normal sEV from the apical membrane, numerous pathways intimately linked with inflammatory processes were identified, including: (i) Inflammatory Disease; (ii) Immunological Disease; and (iii) Organismal Injury and Abnormalities. Additional examination of these apical‐derived inflammatory miRNA/proteins within the “Tox Function” of IPA identified renal‐associated disease pathways, including: (i) Renal Inflammation; and (ii) Renal Nephritis. Further mapping within the IPA “Networks Function” associated 16 of these miRNA/proteins with a cell death and survival/immunological disease pathway (46% of the total molecules in the network) (Figure 4).

**FIGURE 5:**
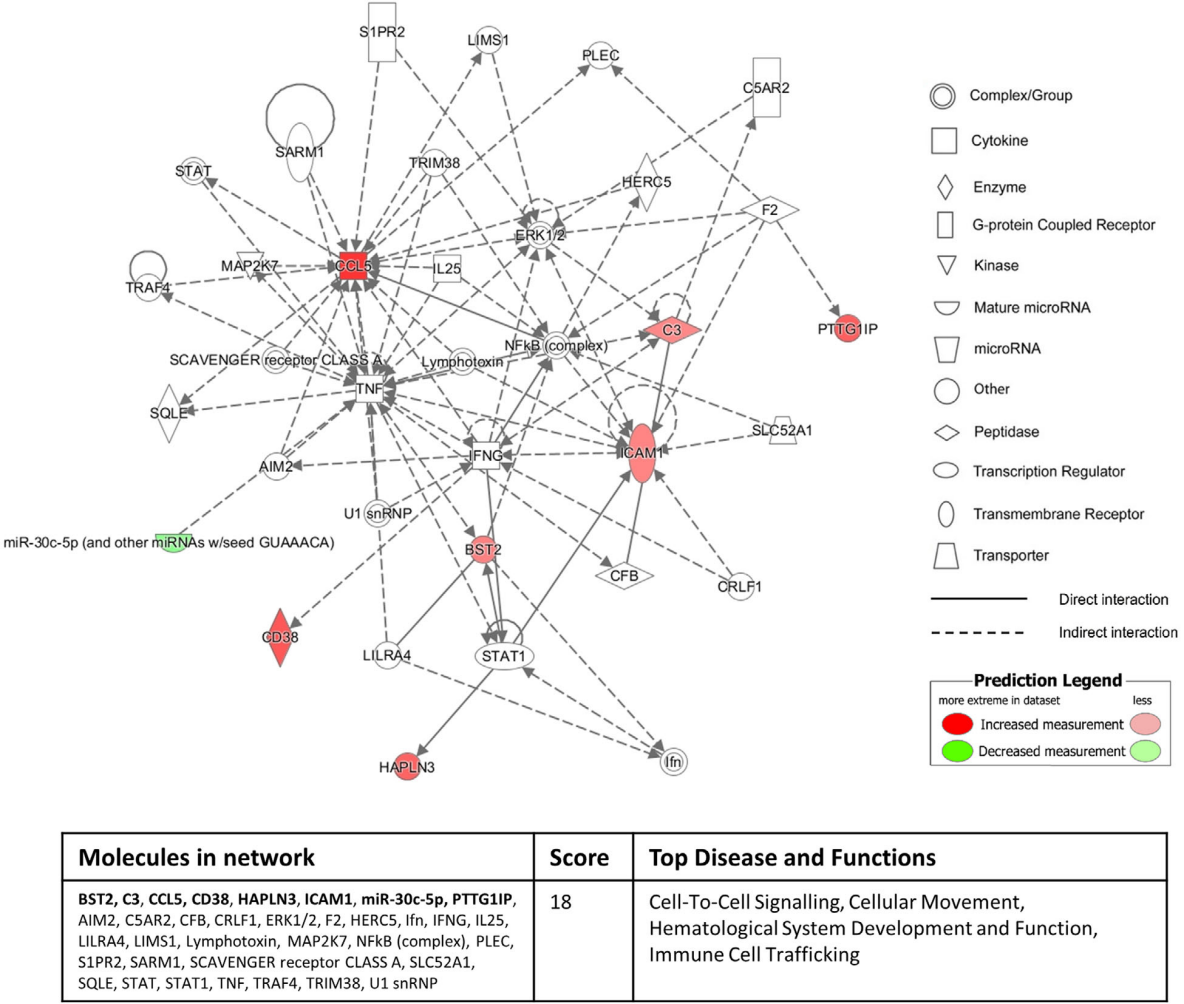
**Eight out of 15 significantly differentially expressed basolateral sEV molecules (defined miRNA/proteins) derived from inflammatory culture conditions compared to normal culture conditions associate with a cell‐to‐cell signalling and immune cell trafficking pathway identified by the “Networks Function” of IPA**. The red colour intensity indicates amount of up‐regulation within the network and the green colour intensity indicates amount of down‐regulation within the network. Molecules highlighted in bold within the data table are the eight significantly differentially basolateral sEV miRNA/proteins.

Equivalent “Disease and Biological Function” clustering of the significantly differentially expressed defined miRNA (4)  and proteins (11)  of inflammatory versus normal sEV from the basolateral membrane revealed pathways of: (i) Immune Cell Trafficking; (ii) Cellular Movement; and (iii) Inflammatory Responses. Further mapping within the “Networks Function” of IPA associated eight out of these 15 miRNA/proteins with a cell‐to‐cell signalling/cellular movement pathway (Figure 5). These network pathway findings suggest that basolateral sEV produced by human primary PTEC under inflammatory conditions may play a functional immuno‐modulatory role in the diseased tubulointerstitial micro‐environment.

### 3.6 Basolateral sEV produced by human primary PTEC under inflammatory diseased conditions initiate and regulate immune cell responses

**FIGURE 6:**
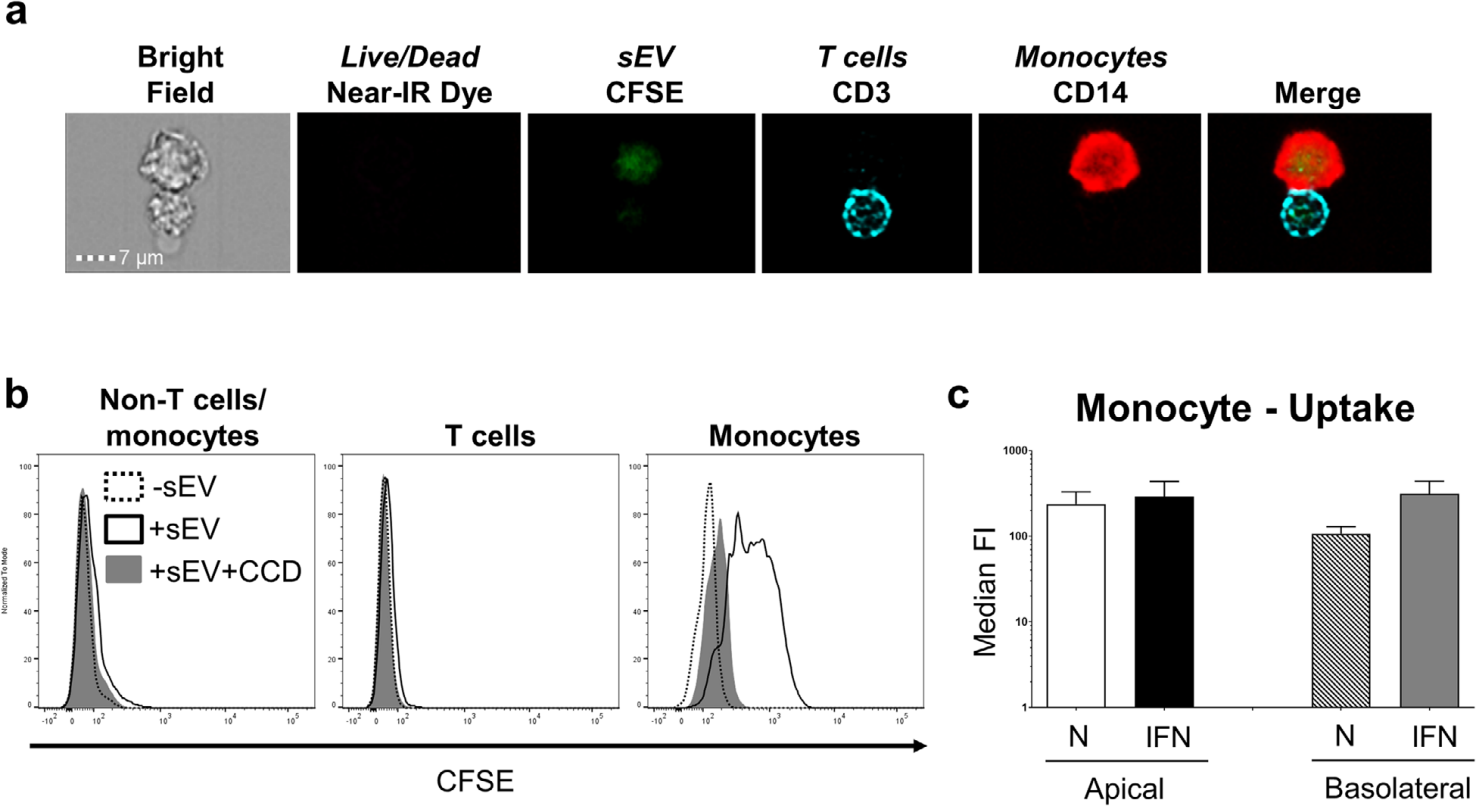
**Equivalent monocyte uptake of apical and basolateral sEV derived from human primary PTEC**. (a) Uptake of CFSE‐labelled, PTEC‐derived apical inflammatory sEV (green) by live (negative for LIVE/DEAD^®^ Fixable Near‐IR Dead Cell reagent) CD3^+^ T cells (aqua) and CD14^+^ monocytes (red) as assessed by imaging flow cytometry. Equivalent images were observed for all apical and basolateral sEV populations. One representative of two PTEC donor experiments. (b) Representative flow cytometric histogram of CFSE signal by live non‐T cells/monocytes (CD3^–^/CD14^–^ PBMC) (left panel), CD3^+^ T cells (middle panel) and live CD14^+^ monocytes (right panel) after culture alone (‐sEV; dashed), or following co‐culture with labelled apical inflammatory sEV in the absence (+sEV; black unfilled) or presence of cytochalasin D (+sEV+CCD; grey filled). Equivalent histograms were observed for all apical and basolateral sEV populations. One representative of five PTEC donor experiments. (c) CFSE signal (median fluorescent intensity; median FI) by live CD14^+^ monocytes following co‐culture with labelled apical or basolateral sEV derived from normal or inflammatory (IFN) PTEC. Results represent mean ± SEM of five individual PTEC donor experiments.

To investigate the functionality of these human PTEC‐derived sEV, we established an *in vitro* immune cell‐sEV co‐culture system. We first monitored uptake of CFSE‐labelled PTEC‐derived sEV by peripheral blood mononuclear cells (PBMC) using imaging and conventional flow cytometry. CFSE‐labelled sEV were efficiently internalized by CD14^+^ monocytes, with no or minimal uptake observed for CD3^+^ T cells and non‐T cells/monocytes (CD3^–^/CD14^–^ PBMC) (Figure 6a,b). The uptake by monocytes was inhibited by cytochalasin D, an inhibitor of actin polymerisation, confirming that sEV were actively internalized rather than attached to the cell surface (Figure 6b). We did not observe any significant differences in monocyte uptake between apical and basolateral sEV populations (Figure 6c).

**FIGURE 7:**
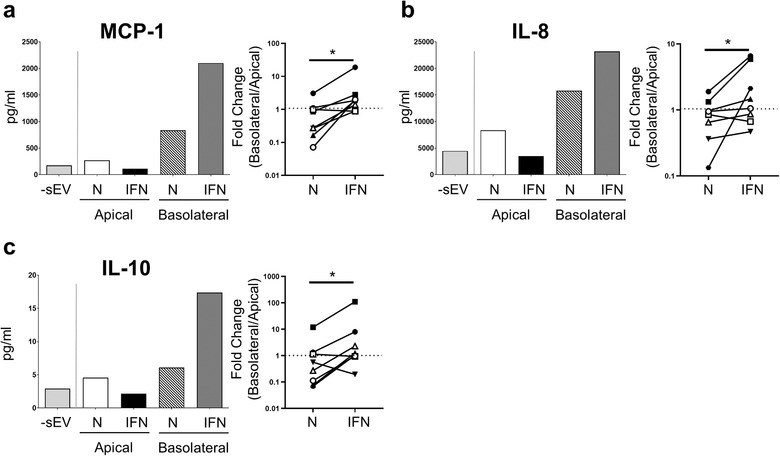
**Basolateral sEV derived from human primary PTEC under inflammatory conditions induce PBMC production of chemokines (MCP‐1, IL‐8) and anti‐inflammatory cytokine IL‐10**. MCP‐1 (a), IL‐8 (b) and IL‐10 (c) production by PBMC following 24 h culture alone (‐sEV) and in the presence of apical or basolateral sEV derived from normal (N) or inflammatory (IFN) PTEC. One representative donor experiment is shown (left panel). Fold change (concentration in response to basolateral sEV/concentration in response to apical sEV; basolateral/apical) in chemokine/cytokine production by PBMC for normal (N) and inflammatory (IFN) PTEC‐derived sEV is shown (right panel); Eight individual donor PTEC experiments are presented, with symbols representing individual donor PTEC experiments. *P < 0.05, Wilcoxon matched‐pairs signed‐rank test.

Chemokine/cytokine production by PBMC in response to PTEC‐derived sEV was also examined (Figure 7). Notably, basolateral sEV isolated from inflammatory conditions induced the highest secretion by PBMC of chemokines monocyte chemoattractant protein (MCP)‐1 and interleukin (IL)‐8 (Figure 7a,b) and immuno‐regulatory cytokine IL‐10 (Figure 7c). Over eight individual co‐culture experiments, the fold change in PBMC production of all three molecules in response to basolateral versus apical sEV (concentration in response to basolateral sEV/concentration in response to apical sEV) was significantly elevated for inflammatory sEV compared with equivalent normal sEV (Figure 7a‐c). These data identify basolateral sEV signalling by human primary PTEC as a novel mechanism for initiating immune cell chemotaxis and regulating cytokine production within the inflammatory diseased tubulointerstitium.

## 4 DISCUSSION

PTEC play a central role in the development and modulation of tubulointerstitial inflammation. We have previously demonstrated that human primary PTEC play an immuno‐regulatory role in the initial stages of kidney injury (i.e., early inflammation) (Kassianos et al., 2013; Sampangi et al., 2015; Sampangi et al., 2015; Wilkinson et al., 2011), but a pathogenic immuno‐stimulatory role in the chronic inflammatory disease state (i.e., tubulointerstitial hypoxia/fibrosis) (Law et al., 2019; Wilkinson et al., 2014). Although prior studies have characterized the polarity‐dependent response of PTEC to injurious stimuli (e.g., apical versus basal membrane expression of surface molecules and cytokine production) (Burton et al., 1996; Lai et al., 2007), our present work is the first to investigate the polarized release and composition of sEV secreted by human primary PTEC under inflammatory conditions. In line with previous sEV/exosome studies of polarized human epithelial cells (e.g., epithelial cells of retinal pigment, intestinal and hepatic origin) (Bu et al., 2010; Davies et al., 2020; Sreekumar et al., 2010), we show, for the first time, a polarized regulation of sEV release/cargo in human primary PTEC. Moreover, we identify a novel mechanism of tubulointerstitial immuno‐regulation mediated by a discrete population of basolateral sEV secreted by inflammatory PTEC.

Polarized PTEC have been identified as the predominant source of tubule‐derived EV in diseased kidneys, with minimal EV production detected in distal tubules and the collecting duct (Liu et al., 2020). A Transwell system has been previously used to model the *in vivo* polarized environment of human PTEC (Burton et al., 1996; Lai et al., 2007; Whitin et al., 2002; Zhao et al., 2017) ‐ our present study extends this approach to examine PTEC sEV biology. We established that human primary PTEC cultured on Transwells under both normal and inflammatory conditions:  (i) develop a confluent monolayer capable of functioning as a diffusion barrier; and (ii)  maintain a stable polarized phenotype as demonstrated by expression profiling of apical protein MRP‐4. These results confirm the suitability of the Transwell system for examining polarized sEV secretion by human primary PTEC.

sEV/exosome biogenesis is regulated by injurious triggers within the extracellular milieu, including inflammation. Increases in exosome numbers have been associated with many acute and chronic non‐neoplastic inflammatory diseases, including sepsis, pre‐eclampsia and atherosclerosis (Im et al., 2020; Salomon et al., 2017; Wang et al., 2019). Elevated numbers of renal and urinary exosomes have also been reported in acute and chronic models of tubulointerstitial inflammation (Lv et al., 2018). Furthermore, our team have demonstrated that total production of sEV/exosomes by human primary PTEC is significantly elevated under inflammatory conditions (Wang et al., 2017). In line with these findings, in our present study, we showed significantly increased numbers of apical and basolateral sEV secreted by human primary PTEC under inflammatory conditions compared with equivalent sEV from normal conditions. Of note, we also identified significantly elevated numbers of apical sEV compared with basolateral sEV under both normal homeostatic and inflammatory conditions. These quantitative differences between apical and basolateral membranes may be reflective of independent pathways of sEV/exosome biogenesis, as proposed in a recent study of polarized exosome release from epithelial cells (Matsui et al., 2020). In this study, Matsui et al. provided evidence of distinct mechanisms of exosome production in polarized cells – that is, apical and basolateral exosome release mediated by ALIX‐Syntenin1‐Syndecan1 and ceramides respectively (Matsui et al., 2020). Further investigation of these mechanisms in human primary PTEC will enable the targeted modulation of sEV production within diseased kidneys.

Urinary EV and their molecular repertoire have been intensely investigated for use as diagnostic non‐invasive biomarkers of inflammatory kidney disorders (Lv et al., 2019). However, the specific cellular origin of urinary EV/molecules is unclear as they may originate from any of the diverse cell types lining the urinary space (e.g., proximal epithelial cells down to urethral‐bladder epithelial cells). In our current study, we provide evidence identifying PTEC (and their apical membrane‐derived sEV released into the tubular lumen) as a potential source of these urinary biomarkers. Of the 12 lipid species significantly up‐regulated in apical sEV derived from inflammatory versus normal PTEC, four ceramide species (Cer d18:1/16:0, Cer d18:1/20:0, Cer d18:1/22:0, Cer d18:1/24:0) have been previously reported as elevated in the urine of diabetic nephropathy patients and correlating with urinary biomarkers of renal tubular injury (e.g., N‐acetyl‐β‐D‐glucosaminidase; NAG) (Morita et al., 2020). Moreover, three of the 14 defined miRNA species significantly elevated in apical sEV derived from inflammatory PTEC (miR‐155‐5p, miR‐132‐3p, miR‐423‐5p) have been implicated in pathways of kidney injury (Lv et al., 2018; Yuan et al., 2017; Zhang et al., 2020). In particular, elevated urinary levels of miR‐155 have been reported in patients with diabetic nephropathy (Beltrami et al., 2018) and IgA nephropathy (Wang et al., 2011), whilst miR‐423 is increased in the urine of AKI patients (Ramachandran et al., 2013). Furthermore, seven of the 20 proteins significantly up‐regulated in apical sEV isolated from inflammatory versus normal PTEC are IFN‐γ and/or TNF‐α‐inducible markers previously identified in studies of kidney tubular cell biology – TNIP1, β2‐microglobulin, IFITM1, ICAM‐1, CXCL10, IDO1, CD40 (Cockwell, 2002; Demmers et al., 2015; Ho et al., 2008; Nugraha et al., 2017; Wilkinson et al., 2011; Yang et al., 2019; Zeng et al., 2014). Of these proteins, urinary β2‐microglobulin, ICAM‐1 and CXCL10 have been reported as non‐invasive biomarkers of proximal tubular injury/tubulointerstitial inflammation (Rabant et al., 2015; Teppo et al., 2001; Zeng et al., 2014). Our molecular profiling of inflammatory PTEC‐derived apical sEV corroborate these previous findings, but also provide additional novel targets for interrogation as urinary biomarkers in human inflammatory kidney diseases.

Our biological pathway analyses of these differentially expressed molecules associated apical inflammatory sEV with processes of cell survival/inflammation. Based on these findings, we propose that inflammatory PTEC‐derived apical sEV released into the urinary space may also play a functional role via signalling to downstream tubular segments. Proximal‐to‐distal sEV/exosomal signalling has emerged as an area of research interest (Lv et al., 2019). An*in vitro* study by Gildea et al. showed uptake of exosomes derived from PTEC lines by both distal tubule and collecting duct cell lines (Gildea et al., 2014). However, the translation of this work to *in vivo* models has yet to be reported. van Balkom et al. have hypothesized that Tamm‐Horsfall protein (uromodulin) may, in fact, capture exosomes within the *in vivo* urinary space and thus, inhibit contact with recipient cells and prevent communication to downstream tubular segments (Van Balkom et al., 2011). Further functional examination of intra‐nephron signalling by PTEC‐derived sEV (in particular, the biological pathways identified in our study) may reveal unique modes of trans‐renal communication.

Equivalent network mapping also associated the molecular repertoire (miRNA/proteins) of basolateral sEV derived from inflammatory PTEC with pathways of immune cell trafficking/cell‐to‐cell signalling. Indeed, PTEC are central players in the initiation of immune cell‐mediated processes within the diseased tubulointerstitium via the release of: (i) inflammatory chemokines/mediators (e.g., MCP‐1/CCL2, IL‐8/CXCL8, CCL5, ICAM‐1); and (ii) complement components (e.g., C3) (Brooimans et al., 1991; Ho et al., 2008; Lai et al., 2007; Tang et al., 2003; Tang et al., 1999; Wang et al., 1997; Zoja et al., 1998). However, the complexities of these PTEC‐immune cell interactions remain to be fully elucidated. We identified significantly elevated CCL5, ICAM‐1 and C3 in basolateral sEV derived from inflammatory PTEC as compared with equivalent normal PTEC. In addition, we reported increased SECTM1 (secreted and transmembrane protein 1) in basolateral inflammatory sEV, previously identified as a chemoattractant for human monocytes in non‐renal diseased tissue (Wang et al., 2014). These established and novel molecules represent putative mechanism/s via which human PTEC‐derived sEV/exosomes may directly initiate tubulointerstitial recruitment/inflammation.

Direct pathways of tubular epithelial cell (TEC) exosome‐mediated inflammation have been previously reported in experimental models of mouse kidney injury, with transfer of exosomal MCP‐1/CCL2 messenger RNA (mRNA) from TEC to macrophages shown to be essential for initiating tubulointerstitial inflammation (Lv et al., 2018). This concept of macrophage internalisation of TEC exosomes is supported by our human data, identifying monocytes as the predominant PBMC population for sEV uptake. Of note, MCP‐1, a chemotactic factor for monocytes/macrophages, was not detected in our molecular profiling of human PTEC‐derived sEV. However, we did observe a significant induction of this chemokine (in addition to neutrophil chemoattractant IL‐8) in PBMC following co‐culture with basolateral sEV derived from inflammatory PTEC. These findings point to a secondary indirect mechanism of PTEC sEV‐mediated inflammation via signalling to immune cells to produce chemokines (MCP‐1/IL‐8) that, in turn, recruit further myeloid cells (monocytes/neutrophils) into the diseased microenvironment.

This initial recruitment of inflammatory immune cells is critical for healthy tissue repair via clearance of apoptotic/necrotic and senescent cells by phagocytes (e.g., macrophages/monocytes and neutrophils) (Docherty et al., 2020). However, if this immune response is not tightly regulated, it can lead to a vicious cycle of persistent and maladaptive inflammation, such as observed in chronic kidney disease. Previous reports have identified tubular epithelial cell‐derived EV as important mediators of anti‐inflammatory events (Feigerlová et al., 2018; Lv et al., 2019), and thus, essential to prevent this pathogenic progression from acute to chronic tubulointerstitial inflammation. Our data support this concept, with a significant induction of IL‐10 protein in PBMC following co‐culture with basolateral inflammatory sEV. IL‐10 is a pluripotent cytokine that has been shown to suppress the progression of kidney injury via its anti‐inflammatory and anti‐apoptotic functions (Sakai et al., 2019). In particular, our team has previously reported that inflammatory PTEC drive IL‐10 production in autologous monocyte‐derived dendritic cells (Kassianos et al., 2013; Sampangi et al., 2015). Further mechanistic dissection of sEV in this PTEC‐mediated immuno‐modulation may offer therapeutic targets for the treatment of kidney diseases.

It is important to also highlight that the concentration of D‐glucose in the PTEC culture medium used in our study (17.5 mM) was higher compared with normoglycemia/normal blood glucose levels (∼5.5 mM). Although this Defined Medium is validated by our and other groups for culture and expansion of human primary PTEC, this increased concentration of D‐glucose could potentially impact the response of cells to normal and inflammatory conditions and is a notable factor for consideration when interpreting results of this study.

Collectively, these results provide the first comprehensive molecular and functional characterisation of polarity‐dependent sEV secretion by human primary PTEC. We provide evidence that the molecular cargo of PTEC‐derived apical sEV are promising urinary biomarker candidates for inflammatory kidney diseases. Notably, we also identified significantly elevated ceramide species (e.g., Cer d18:1/18:0, Cer d18:1/24:1) in basolateral inflammatory sEV that have been recently reported as increased in the circulation (plasma) of patients with chronic kidney disease (Mantovani et al., 2020) and may be of diagnostic utility in future validation studies. We also offer unique insights into the biological pathways through which polarized human PTEC mediate proximal‐to‐distal signalling (via apical sEV) and tubular‐immune cell communication (via basolateral sEV). The broader application of these findings will enable the development of novel approaches with greater therapeutic specificity for maintaining the appropriate balance between pro‐ and anti‐inflammatory processes in human kidney diseases.

## CONFLICT OF INTEREST

The authors report no conflicts of interest.

## Supporting information


**Supplementary Figure 1**. (a‐b) Immunofluorescent microscopy of normal (a) and inflammatory (b) PTEC monolayers stained for β‐tubulin (green), MRP‐4 (red) and DAPI (blue). Basolateral (bottom) to apical (top) expression of MRP‐4 is presented in a Z‐stack image series. Scale bars represent 20μm. One representative of three PTEC donor experiments.

**Supplementary Table 1**. Clinical and histological features of PTEC donors at the time of nephrectomy.

**Supplementary Table 2**. Significantly differentially expressed sEV lipid species.

**Supplementary Table 3**. Significantly differentially expressed sEV novel miRNA.

**Supplementary Table 4**. Significantly differentially expressed sEV defined miRNA.

SUPPORTING INFORMATION
