# Supplementary figures and images for: Molecular and functional profiling of apical versus basolateral small extracellular vesicles derived from primary human proximal tubular epithelial cells under inflammatory conditions
Source: J Extracell Vesicles. 2021 Feb 16;10(4):e12064. doi: 10.1002/jev2.12064 (PMC7886702; doi:10.1002/jev2.12064)

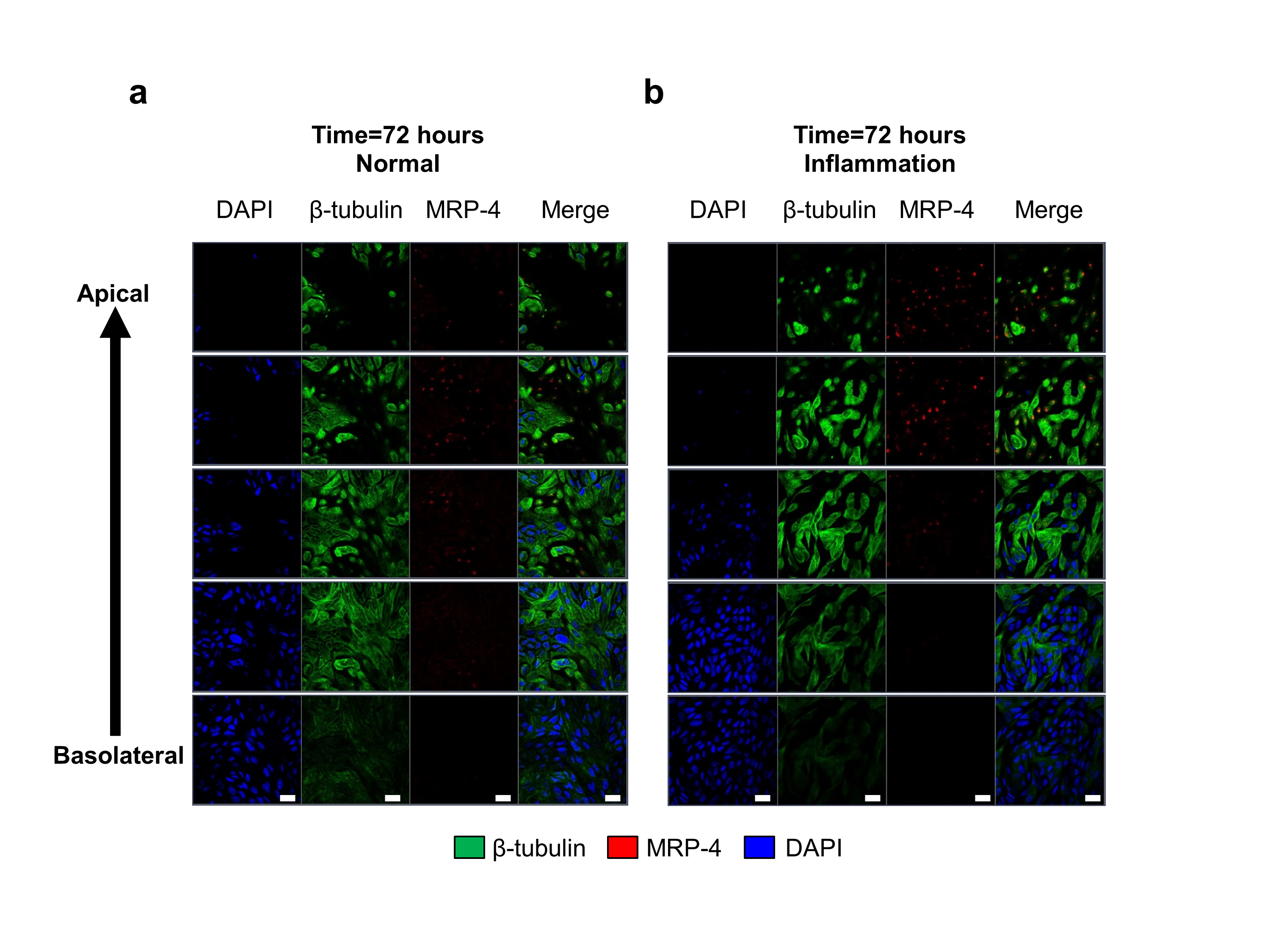

Supplement: Supplementary file 1 — Supplementary Figure 1. (a‐b) Immunofluorescent microscopy of normal (a) and inflammatory (b) PTEC monolayers stained for β‐tubulin (green), MRP‐4 (red) and DAPI (blue). Basolateral (bottom) to apical (top) expression of MRP‐4 is presented in a Z‐stack image series. Scale bars represent 20μm. One representative of three PTEC donor experiments. [file JEV2-10-e12064-s001.TIF]

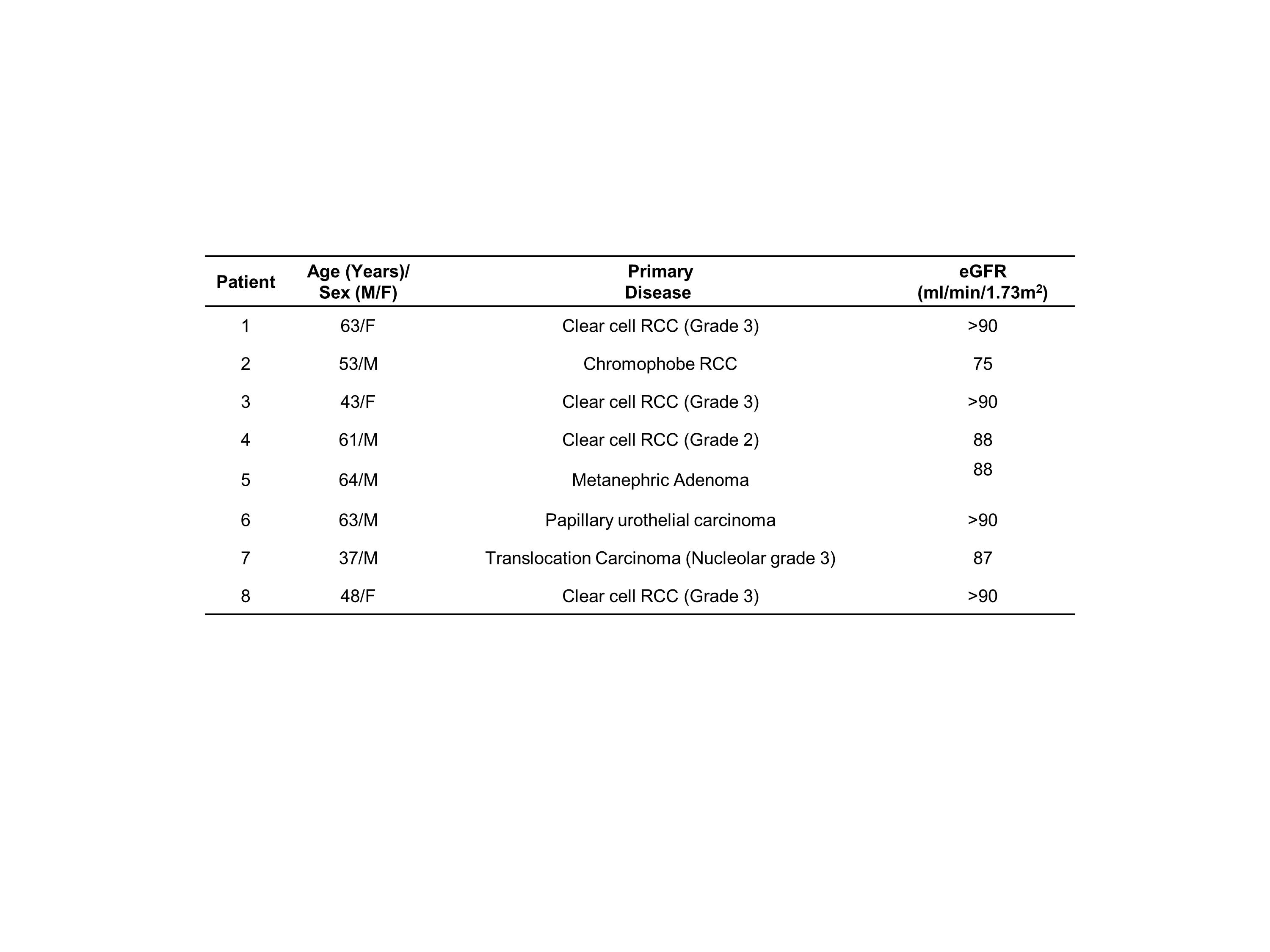

Supplement: Supplementary file 2 — Supplementary Table 1. Clinical and histological features of PTEC donors at the time of nephrectomy. [file JEV2-10-e12064-s002.TIF]

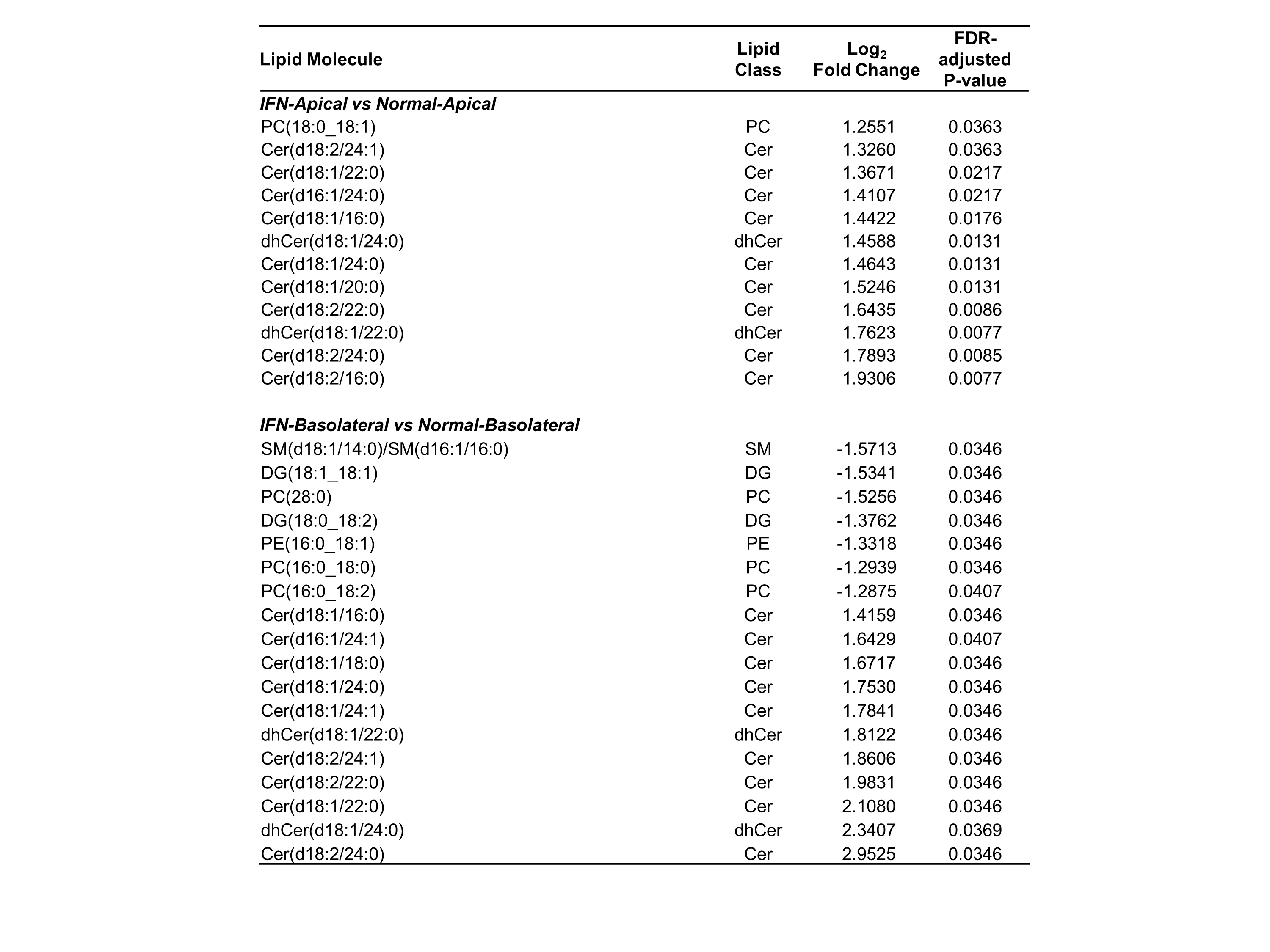

Supplement: Supplementary file 3 — Supplementary Table 2. Significantly differentially expressed sEV lipid species. [file JEV2-10-e12064-s003.TIF]

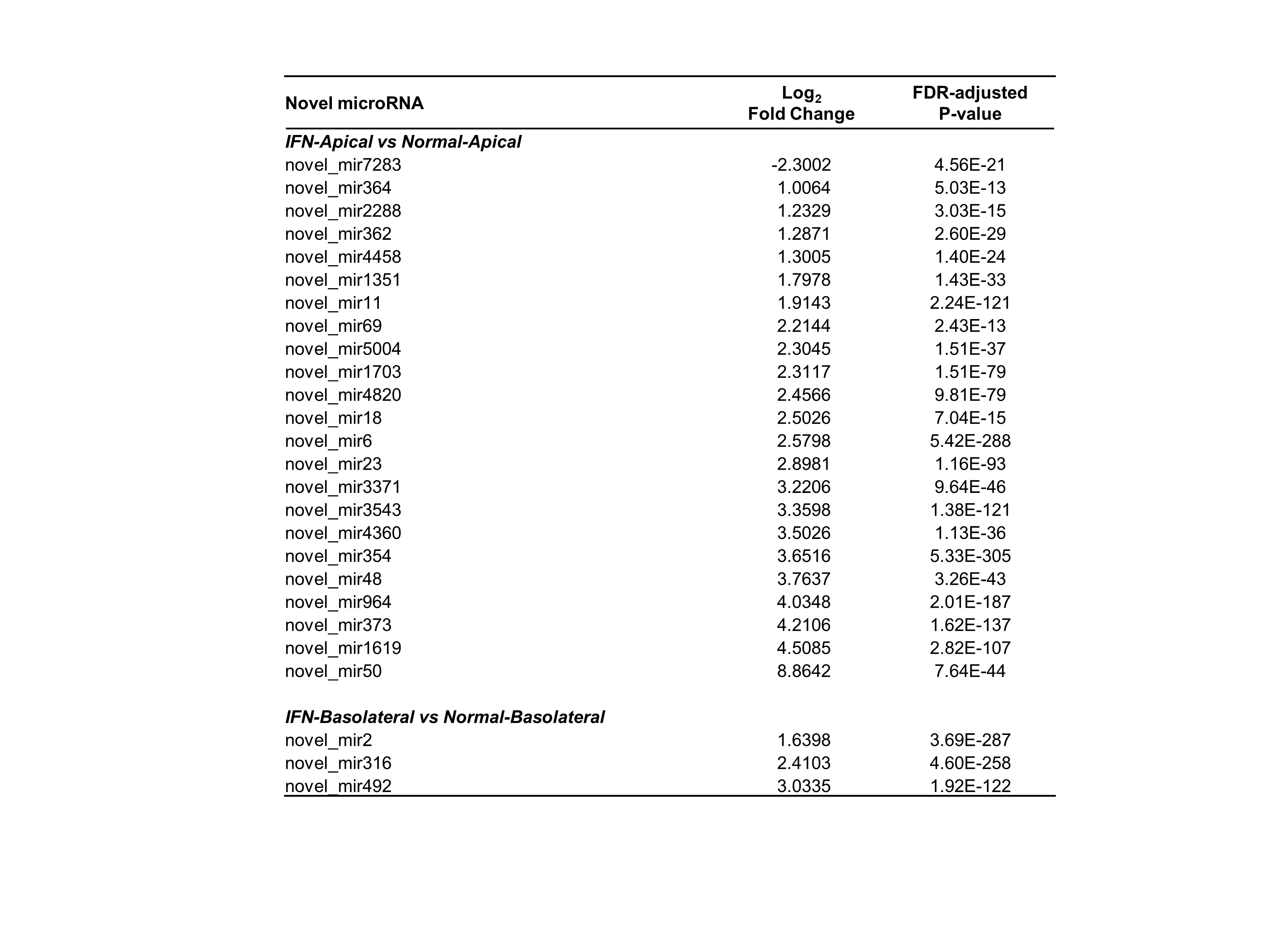

Supplement: Supplementary file 4 — Supplementary Table 3. Significantly differentially expressed sEV novel miRNA. [file JEV2-10-e12064-s004.TIF]

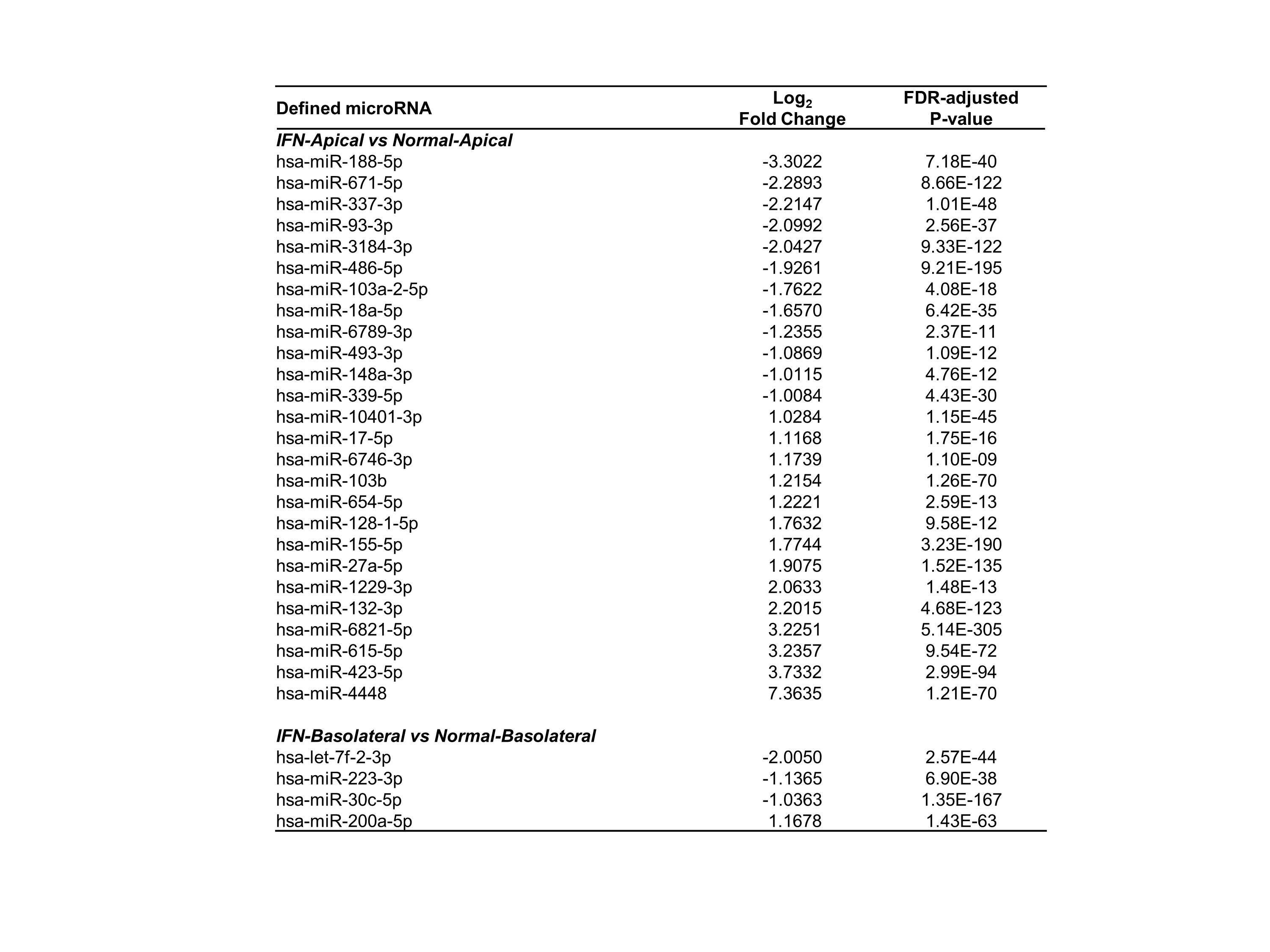

Supplement: Supplementary file 5 — Supplementary Table 4. Significantly differentially expressed sEV defined miRNA. [file JEV2-10-e12064-s005.TIF]
