# Supplementary material for: Molecular and functional profiling of apical versus basolateral small extracellular vesicles derived from primary human proximal tubular epithelial cells under inflammatory conditions
Source: J Extracell Vesicles. 2021 Feb 16;10(4):e12064. doi: 10.1002/jev2.12064 (PMC7886702; doi:10.1002/jev2.12064)
Supplement: Supplementary file 6 — SUPPORTING INFORMATION [file JEV2-10-e12064-s006.docx]

**SUPP TABLE 1**

| **Patient** | **Age (Years)/**  **Sex (M/F)** | **Primary**  **Disease** | **eGFR**  **(ml/min/1.73m^2^)** |
| --- | --- | --- | --- |
| 1 | 63/F | Clear cell RCC (Grade 3) | >90 |
| 2 | 53/M | Chromophobe RCC | 75 |
| 3 | 43/F | Clear cell RCC (Grade 3) | >90 |
| 4 | 61/M | Clear cell RCC (Grade 2) | 88 |
| 5 | 64/M | Metanephric Adenoma | 88 |
| 6 | 63/M | Papillary urothelial carcinoma | >90 |
| 7 | 37/M | Translocation Carcinoma (Nucleolar grade 3) | 87 |
| 8 | 48/F | Clear cell RCC (Grade 3) | >90 |

**SUPP TABLE 2**

| **Lipid Molecule** | **Lipid**  **Class** | **Log_2_**  **Fold Change** | **FDR-adjusted**  **P-value** |
| --- | --- | --- | --- |
| ***IFN-Apical vs Normal-Apical*** |  |  |  |
| PC(18:0_18:1) | PC | 1.2551 | 0.0363 |
| Cer(d18:2/24:1) | Cer | 1.3260 | 0.0363 |
| Cer(d18:1/22:0) | Cer | 1.3671 | 0.0217 |
| Cer(d16:1/24:0) | Cer | 1.4107 | 0.0217 |
| Cer(d18:1/16:0) | Cer | 1.4422 | 0.0176 |
| dhCer(d18:1/24:0) | dhCer | 1.4588 | 0.0131 |
| Cer(d18:1/24:0) | Cer | 1.4643 | 0.0131 |
| Cer(d18:1/20:0) | Cer | 1.5246 | 0.0131 |
| Cer(d18:2/22:0) | Cer | 1.6435 | 0.0086 |
| dhCer(d18:1/22:0) | dhCer | 1.7623 | 0.0077 |
| Cer(d18:2/24:0) | Cer | 1.7893 | 0.0085 |
| Cer(d18:2/16:0) | Cer | 1.9306 | 0.0077 |
|  |  |  |  |
| ***IFN-Basolateral vs Normal-Basolateral*** |  |  |  |
| SM(d18:1/14:0)/SM(d16:1/16:0) | SM | -1.5713 | 0.0346 |
| DG(18:1_18:1) | DG | -1.5341 | 0.0346 |
| PC(28:0) | PC | -1.5256 | 0.0346 |
| DG(18:0_18:2) | DG | -1.3762 | 0.0346 |
| PE(16:0_18:1) | PE | -1.3318 | 0.0346 |
| PC(16:0_18:0) | PC | -1.2939 | 0.0346 |
| PC(16:0_18:2) | PC | -1.2875 | 0.0407 |
| Cer(d18:1/16:0) | Cer | 1.4159 | 0.0346 |
| Cer(d16:1/24:1) | Cer | 1.6429 | 0.0407 |
| Cer(d18:1/18:0) | Cer | 1.6717 | 0.0346 |
| Cer(d18:1/24:0) | Cer | 1.7530 | 0.0346 |
| Cer(d18:1/24:1) | Cer | 1.7841 | 0.0346 |
| dhCer(d18:1/22:0) | dhCer | 1.8122 | 0.0346 |
| Cer(d18:2/24:1) | Cer | 1.8606 | 0.0346 |
| Cer(d18:2/22:0) | Cer | 1.9831 | 0.0346 |
| Cer(d18:1/22:0) | Cer | 2.1080 | 0.0346 |
| dhCer(d18:1/24:0) | dhCer | 2.3407 | 0.0369 |
| Cer(d18:2/24:0) | Cer | 2.9525 | 0.0346 |

**SUPP TABLE 3**

| **Novel microRNA** | **Log_2_**  **Fold Change** | **FDR-adjusted**  **P-value** |
| --- | --- | --- |
| ***IFN-Apical vs Normal-Apical*** |  |  |
| novel_mir7283 | -2.3002 | 4.56E-21 |
| novel_mir364 | 1.0064 | 5.03E-13 |
| novel_mir2288 | 1.2329 | 3.03E-15 |
| novel_mir362 | 1.2871 | 2.60E-29 |
| novel_mir4458 | 1.3005 | 1.40E-24 |
| novel_mir1351 | 1.7978 | 1.43E-33 |
| novel_mir11 | 1.9143 | 2.24E-121 |
| novel_mir69 | 2.2144 | 2.43E-13 |
| novel_mir5004 | 2.3045 | 1.51E-37 |
| novel_mir1703 | 2.3117 | 1.51E-79 |
| novel_mir4820 | 2.4566 | 9.81E-79 |
| novel_mir18 | 2.5026 | 7.04E-15 |
| novel_mir6 | 2.5798 | 5.42E-288 |
| novel_mir23 | 2.8981 | 1.16E-93 |
| novel_mir3371 | 3.2206 | 9.64E-46 |
| novel_mir3543 | 3.3598 | 1.38E-121 |
| novel_mir4360 | 3.5026 | 1.13E-36 |
| novel_mir354 | 3.6516 | 5.33E-305 |
| novel_mir48 | 3.7637 | 3.26E-43 |
| novel_mir964 | 4.0348 | 2.01E-187 |
| novel_mir373 | 4.2106 | 1.62E-137 |
| novel_mir1619 | 4.5085 | 2.82E-107 |
| novel_mir50 | 8.8642 | 7.64E-44 |
|  |  |  |
| ***IFN-Basolateral vs Normal-Basolateral*** |  |  |
| novel_mir2 | 1.6398 | 3.69E-287 |
| novel_mir316 | 2.4103 | 4.60E-258 |
| novel_mir492 | 3.0335 | 1.92E-122 |

**SUPP TABLE 4**

| **Defined microRNA** | **Log_2_**  **Fold Change** | **FDR-adjusted**  **P-value** |
| --- | --- | --- |
| ***IFN-Apical vs Normal-Apical*** |  |  |
| hsa-miR-188-5p | -3.3022 | 7.18E-40 |
| hsa-miR-671-5p | -2.2893 | 8.66E-122 |
| hsa-miR-337-3p | -2.2147 | 1.01E-48 |
| hsa-miR-93-3p | -2.0992 | 2.56E-37 |
| hsa-miR-3184-3p | -2.0427 | 9.33E-122 |
| hsa-miR-486-5p | -1.9261 | 9.21E-195 |
| hsa-miR-103a-2-5p | -1.7622 | 4.08E-18 |
| hsa-miR-18a-5p | -1.6570 | 6.42E-35 |
| hsa-miR-6789-3p | -1.2355 | 2.37E-11 |
| hsa-miR-493-3p | -1.0869 | 1.09E-12 |
| hsa-miR-148a-3p | -1.0115 | 4.76E-12 |
| hsa-miR-339-5p | -1.0084 | 4.43E-30 |
| hsa-miR-10401-3p | 1.0284 | 1.15E-45 |
| hsa-miR-17-5p | 1.1168 | 1.75E-16 |
| hsa-miR-6746-3p | 1.1739 | 1.10E-09 |
| hsa-miR-103b | 1.2154 | 1.26E-70 |
| hsa-miR-654-5p | 1.2221 | 2.59E-13 |
| hsa-miR-128-1-5p | 1.7632 | 9.58E-12 |
| hsa-miR-155-5p | 1.7744 | 3.23E-190 |
| hsa-miR-27a-5p | 1.9075 | 1.52E-135 |
| hsa-miR-1229-3p | 2.0633 | 1.48E-13 |
| hsa-miR-132-3p | 2.2015 | 4.68E-123 |
| hsa-miR-6821-5p | 3.2251 | 5.14E-305 |
| hsa-miR-615-5p | 3.2357 | 9.54E-72 |
| hsa-miR-423-5p | 3.7332 | 2.99E-94 |
| hsa-miR-4448 | 7.3635 | 1.21E-70 |
|  |  |  |
| ***IFN-Basolateral vs Normal-Basolateral*** |  |  |
| hsa-let-7f-2-3p | -2.0050 | 2.57E-44 |
| hsa-miR-223-3p | -1.1365 | 6.90E-38 |
| hsa-miR-30c-5p | -1.0363 | 1.35E-167 |
| hsa-miR-200a-5p | 1.1678 | 1.43E-63 |
